# Simulating the exchange of Majorana zero modes with a photonic system

**DOI:** 10.1038/ncomms13194

**Published:** 2016-10-25

**Authors:** Jin-Shi Xu, Kai Sun, Yong-Jian Han, Chuan-Feng Li, Jiannis K. Pachos, Guang-Can Guo

**Affiliations:** 1 Key Laboratory of Quantum Information, Department of Optics and Optical Engineering, University of Science and Technology of China, CAS, JinZhai Road 96, Hefei 230026, China; 2 Synergetic Innovation Center of Quantum Information and Quantum Physics, University of Science and Technology of China, Hefei, Anhui 230026, China; 3 School of Physics and Astronomy, University of Leeds, Leeds LS2 9JT, UK

## Abstract

The realization of Majorana zero modes is in the centre of intense theoretical and experimental investigations. Unfortunately, their exchange that can reveal their exotic statistics needs manipulations that are still beyond our experimental capabilities. Here we take an alternative approach. Through the Jordan–Wigner transformation, the Kitaev's chain supporting two Majorana zero modes is mapped to the spin-1/2 chain. We experimentally simulated the spin system and its evolution with a photonic quantum simulator. This allows us to probe the geometric phase, which corresponds to the exchange of two Majorana zero modes positioned at the ends of a three-site chain. Finally, we demonstrate the immunity of quantum information encoded in the Majorana zero modes against local errors through the simulator. Our photonic simulator opens the way for the efficient realization and manipulation of Majorana zero modes in complex architectures.

## Abstract (web summary)

Majorana zero modes are a potential resource for quantum information processing as they offer immunity to noise, but they are difficult to create and control experimentally. Here, the authors use a photonic quantum simulator to mimic the exchange of Majorana zero modes in a spin-half chain.

## Graphical abstract

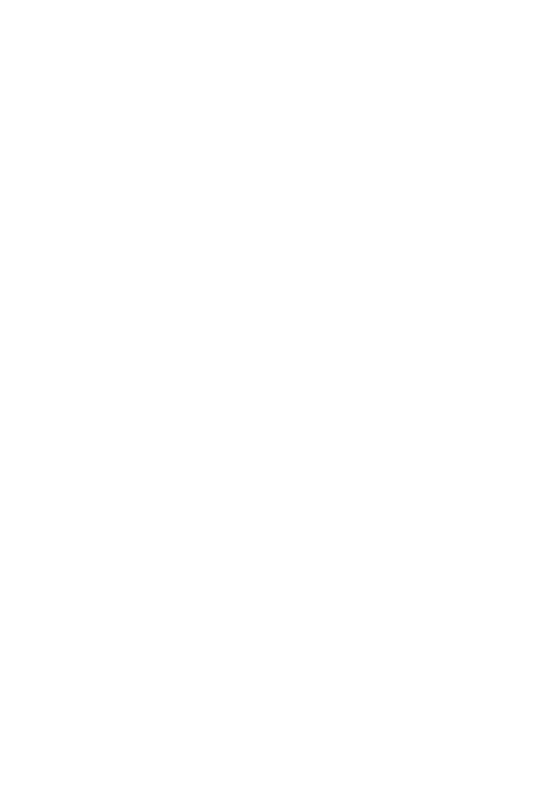

Majorana zero modes (MZMs) give rise to a non-trivial Hilbert space encoded in their degenerate states. When two MZMs are exchanged, their degenerate subspace can be transformed by a unitary operator that signals their non-Abelian statistics^1^. In addition, quantum information encoded in the degenerate subspace of MZMs is naturally immune to local errors. The degeneracy of these states is protected by the fermion parity symmetry; thus, it cannot be lifted by any local symmetry-preserving perturbation. These unique characteristics make MZMs of interest to fundamental physics, whereas they can potentially provide novel and powerful methods for quantum information storing and processing^2^.

The investigation of MZMs has attracted great attention. Rapid theoretical developments^3^,^4^,^5^ have greatly simplified the technological requirements and made it possible to experimentally observe MZMs. A few positive signatures of the formation of MZMs have been reported recently in solid-state systems^6^,^7^,^8^,^9^,^10^,^11^. Nevertheless, the central characteristic of exotic statistics requires the trapping and transport of MZMs, to perform their exchange. This level of manipulation of MZMs is beyond our current control of solid-state systems.

In the following, we overcome this hurdle by taking advantage of the quantum simulation approach^12^: although the simulated system is not experimentally accessible with the current technology, the quantum simulator and its measurement results provide information about the simulated system. We use a photonic quantum simulator to investigate the exchange of MZMs supported in the one-dimensional Kitaev Chain Model (KCM). In principle, we can directly map the Fock space of the Majorana system to the space of the quantum simulator. Nevertheless, to make our approach easily accessible to other scalable systems, such as trapped ions^13^ or Josephson junctions^14^, where spin modes are directly available, we divide the map into two consecutive steps. First, we perform the mapping of the Majorana system to the spin-1/2 system via the Jordan–Wigner (JW) transformation^15^,^16^,^17^,^18^. Next, we perform the mapping of the spin system to the spatial modes of single photons. The JW transformation^19^ is a non-local transformation that relates the states and evolutions of the topological superconducting Kitaev chain to the states and evolutions of a spin-1/2 chain. The corresponding spin system can then be simulated by our optical simulator. The simulated system has only two Majoranas (separated by a gap from all other excitations), any adiabatic manipulation can only result in a U(1) × U(1) transformation that carries the hallmark of their anyonic statistics. In this way, we are able to demonstrate the exchange of two MZMs in a three-site Kitaev chain encoded in the spatial modes of photons. We further demonstrate that quantum information encoded in the degenerate ground state is immune to local phase and flip noise errors.

Owing to the JW transformation, some of the non-local properties of the MZMs are lost in the simulated spin system. Indeed, local single-particle measurements can reveal the fusion space of the corresponding MZMs, something that is not possible in the fermionic picture. At the same time, some of the local properties of the MZMs are also lost. For example, two isolated Majorana fermions, located at the end points of the chain, correspond to excitons in the spin system that are spread along the whole chain. Nevertheless, as the spectra of the two systems are the same, their corresponding quantum evolutions are equivalent. This gives us the means to investigate the statistical behaviour of the MZMs as well as their resilience to errors. Our current simulation only probes the Abelian part of the exchange, as it employs two isolated MZMs. However, our technology and methodology can be used to demonstrate the non-Abelian statistics of Majorana fermions in which four MZMs are needed. This establishes our photonic quantum simulator as a novel platform to study the exotic physics of Majorana fermions and their possible applications to quantum information^20^. Thus, our results extend the capabilities of optical simulators.

## Results

### Majorana braiding

To clearly illustrate the properties of MZMs, we first consider the fermionic KCM^5^. This is the simplest model that supports two MZMs, *γ*_A_ and *γ*_B_ at its boundaries and exhibits twofold degeneracy in its ground state. The braiding properties of Majorana fermions can be investigated by exchanging two MZMs. To perform such an exchange, an adiabatic evolution between carefully constructed Hamiltonians (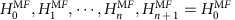) have been proposed to probe the statistics of the MZMs in the KCM^21^,^22^. The exchange of *γ*_A_ and *γ*_B_ induces the braiding evolution 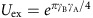 (ref. 21) acting on the twofold-degenerate ground-state space. As the MZMs belong to the same chain with a fixed fusion channel, the braiding matrix is diagonal. Its elements are given in terms of geometric phases^23^ on the basis of |0_Lf_〉 (odd fermion parity) and |1_Lf_〉 (even fermion parity), where the subscript f denotes fermionic states and L is the number of sites of the chain (see Supplementary Note 1). According to the work of Pancharatnam^24^ and the Bargmann invariants^25^, the geometric phases resulting from the braiding process can be directly determined through projective measurements


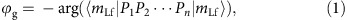


where *ϕ*_g_ is the geometric phase associated with |*m*_Lf_〉, which represent the basis for the ground states of the Hamiltonian 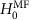 (*m*=0 or 1). Here, *P*_*j*_ projects the system into the ground space of 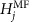, for *j*=1,...,*n*. The geometric phases are gauge invariant and uniquely determined by the Hamiltonians 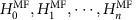, whereas the details of the adiabatic processes between them are not essential^26^,^27^. In general, the projective measurement can be expressed as an imaginary-time evolution (ITE) operator 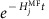 with a sufficiently large evolution time *t* (ref. 28). The adiabatic requirement preserves the fermion parity of the initial state and guarantees that the off-diagonal elements in *U*_ex_ remain zero^26^. Therefore, the whole information of *U*_ex_ can be read out from ITE operators 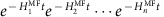 (ref. 29). By employing the method of dissipative evolution^30^, each projective measurement can be efficiently completed with some finite probability (a general circuit is given in Supplementary Note 2). We shall employ this method to experimentally probe the geometric phases obtained during the exchange of two MZMs by simulating a spin model that is equivalent to the KCM.

**Figure 1:**
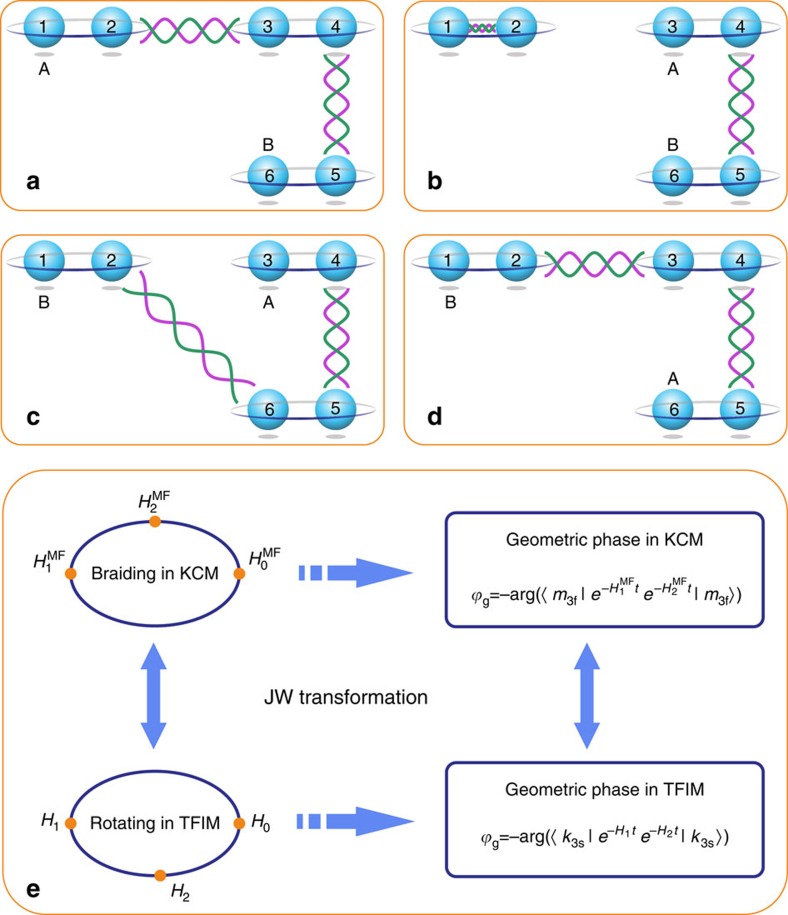
The braiding of Majorana zero modes and the mapping between the fermionic and spin models. The spheres with numbers at their centres represent the Majorana fermions, *γ*_*j*_, for *j*=1,…,6, at the corresponding sites. A pair of Majorana fermions bounded by an enclosing ring represents a normal fermion. The wavy lines between different sites represent the interactions between them. The interactions illustrated in **a**,**b**,**c** and **d** represent the Hamiltonians 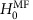, 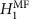, 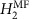 and 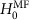, respectively. The letters A and B in each pane represent the corresponding isolated Majorana zero modes. The mapping between the Kitaev chain model (KCM) and the transverse-field Ising model (TFIM) through the JW transformation is shown in **e**. *m*_3f_ and *k*_3s_ are bases of the ground-state spaces defined in the KCM and TFIM (*m* and *k*=0 or 1), respectively.

To complete the exchanging of two MZMs, a four-fermion scheme has been proposed in the superconducting wire system^21^, which is further reduced to a three-fermion scheme in our work. The three-fermion KCM can be described in terms of six Majorana fermions, denoted by *γ*_*j*_ (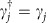, 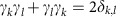 for 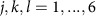), as shown in Fig. 1. The exchanging process can be completed by a set of adiabatic processes among three different Hamiltonians, as illustrated in Fig. 1a–d. The initial Hamiltonian is 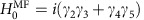. The MZMs *γ*_1_ and *γ*_6_ are isolated at the boundary of the chain to form two MZMs, namely A and B (see Fig. 1a), and the degenerate ground-state space of 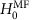 is spanned by |0_3f_〉 and |1_3f_〉 (L=3). The other two Hamiltonians are as follows: 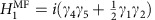, where *γ*_3_ and *γ*_6_ are isolated to form MZMs (see Fig. 1b), and 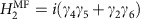, where *γ*_1_ and *γ*_3_ are isolated to form MZMs (see Fig. 1c). The MZMs cross during the adiabatic transition from 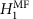 to 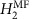. To complete the exchange process, we adiabatically transform 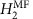 back into 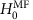 and the Majorana mode A is driven to the location of *γ*_6_ (see Fig. 1d). The exchange of A and B is thus completed, introducing the unitary operation 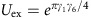 in the ground-state space on the basis of |0_3f_〉 and |1_3f_〉.

Through the JW transformation, a general KCM can be transformed into a one-dimensional transverse-field Ising model (TFIM)^15^. However, these two models have some differences in their physics (see Supplementary Note 3). In the fermionic system, the total fermion parity is fixed. The braiding effect in a single KCM is an overall phase. This phase cannot be directly measured as the superposition state with different fermionic parity is impossible. Despite that, the state of the spin model can be prepared in any superposition state. Hence, the relative geometric phase obtained during the exchange can be measured. As the KCM and the TFIM in the ferromagnetic region have the same spectra and their corresponding quantum evolutions are equivalent, the geometric phases obtained from the braiding evolution *U*_ex_ are invariant under this mapping (see Supplementary Note 4). As a result, the well-controlled spin system offers a good platform to determine the exchange matrix and investigate the statistical behaviour of MZMs.

The Hamiltonians involved in the three-fermion braiding scheme of the KCM, that is, 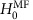, 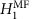 and 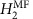, are transformed through the JW transformation into a spin-1/2 system as follows (see Supplementary Note 5 for more details):


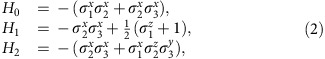


where 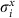, 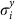 and 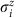 are Pauli matrices of the *i*th spin. The ground states of these Hamiltonians are twofold degenerated. The basis of the ground states of *H*_0_ are denoted by |0_3s_〉 and |1_3s_〉, (s indicates spin states) corresponding to the basis of |0_3f_〉 and |1_3f_〉 in KCM through JW transformation, respectively. The detailed form of 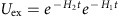 in the ground space of *H*_0_, which is the same as 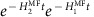 in the ground space of 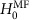, can be obtained by the tomography process, where the initial ground states of *H*_0_ are obtained from a randomly chosen input state |*φ*_r_〉 by implementing 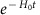 with a sufficiently large *t*, that is, 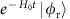 (not normalized). The relative geometric phases and the exchange property of MZMs in the corresponding KCM can then be deduced from the information of *U*_ex_. The mapping between the three-particle KCM and TFIM is further shown in Fig. 1e.

### Local noise immunity

The robustness of the information encoded in the ground space of the KCM with Hamiltonian 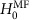 can also be studied in our system. We note that the degeneracy of the ground states of *H*_0_ is not topologically protected, as it can be lifted by a local operator that breaks Z_2_ symmetry, whereas the ground state of KCM is topologically protected^31^. However, owing to the equivalence of the spectra of the 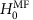 and *H*_0_, the information encoded in the ground state of *H*_0_ is protected by the same excitation gap against local noises with Z_2_ symmetry, which correspond to the noises in fermionic system. It is worth noting that, in our work, the ground states obtained from the ITE process are algorithmically encoded similar to that in quantum error correction^32^. This non-local encoding of states in a larger number of physical qubits protects them against local flip and phase errors. In other words, the Ising model is a good memory with some of the robustness characteristics of the Majorana^32^. As a result, the immunity of the local noises in KCM can be faithfully investigated, which establishes it as an attractive medium for storing and processing quantum information^15^.

Without loss of generality, we take the local error *D*_*j*_ acting on a certain site *j* of the KCM. To verify the robustness against this local error, we should confirm that the operator *D*_*j*_ has a trivial effect on any state in the ground-state space of 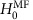, that is, 〈*φ*_f_|*D*_*j*_|*φ*_f_〉 is constant for all the states |*φ*_f_〉 in the ground-state space of 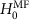. There are two independent types of local noise: flip error (*X*_*j*_) and phase error (*Z*_*j*_). According to the argument presented in ref. 5, the single local flip error is impossible due to the fermionic parity conversation in the ideal KCM (we do not consider the quasiparticle poisoning effect in practical systems^33^). Therefore, the local flip error can only happen at two sites simultaneously, such as 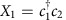 (*c*_*j*_ and 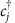 are the annihilation and creation operators of the *j*th fermions, respectively). In terms of the TFIM the *X*_1_ error operator becomes 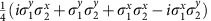 through the JW transformation. Meanwhile, the local phase error on site 1, 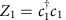, corresponds to the operator 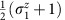 of the spin system. Hence, to confirm the robustness of local errors in KCM, we need to check that 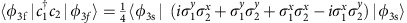 and 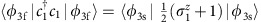 are constant for all states |*φ*_3s_〉 in the ground-state space of *H*_0_ (corresponding to all states |*φ*_3f_〉 in the ground state space of 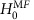).

### Experimental results on simulating the braiding evolution

In our experiment the ITE operator plays a central role. For our current purpose, a useful method to implement the ITE is to design appropriate dissipative evolution^30^, in which the ground-state information of the corresponding Hamiltonian is preserved but the information of the other states is dissipated (see Methods). Taking advantage of the commutation between 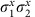 and 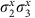, 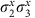 and 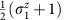, and 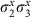 and 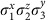 in equation (2), the operator 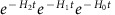 can be expressed as


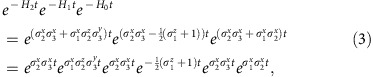


where we set *t*=5 in our analysis. Owing to the fact that the eigenvectors and eigenvalues of each local operator, such as 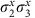, can be obtained easily, the operation of 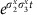 can be implemented by the basis rotation (local unitary gate operation) and dissipation straightforwardly (see Supplementary Note 6 for more details). We therefore introduce an environmental degree of freedom and couple it to the system, which can be simply written as 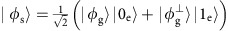, where 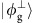 denotes the states that are orthogonal to the ground state |*φ*_g_〉. The environmental basis of |1_e_〉 is dissipated during the evolution and only |0_e_〉 is preserved. Therefore, the ground state of the corresponding Hamiltonian is obtained in the digital quantum simulator^34^. It is worth to emphasize that this process is only dependent on the Hamiltonian. The general algorithm of ITE for a general Hamiltonian has been demonstrated in investigating algorithmic quantum cooling^35^.

Here we employ ITE in optical systems, which provide ideal platforms for quantum simulations. They offer good control of their external parameters and they are largely isolated by their environment^36^. As a proof-of-principle demonstration we map the three-fermion system to the spin model and encode their information in spatial modes. In this way we can experimentally probe the braiding matrix in an all-optical system.

**Figure 2:**
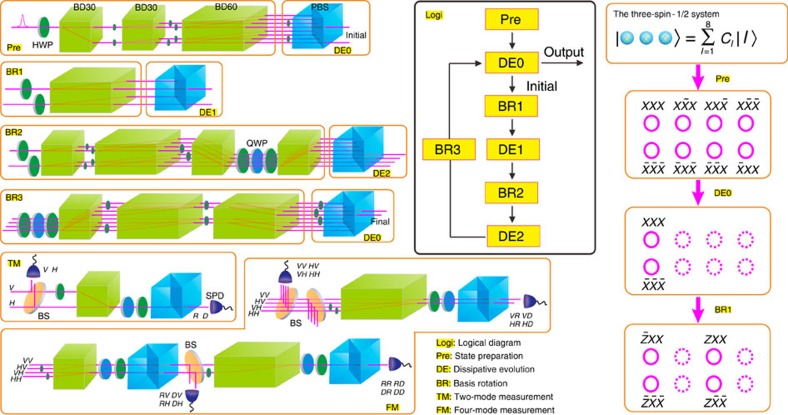
Experimental setup for the simulation of the exchange of Majorana zero modes. The process follows the logical diagram provided in the pane enclosed by the black solid line, denoted by Logi. The state of the three-spin-1/2 system can be expressed in the eight-dimensional space with the basis denoted by |*l*〉 (*l*=1 to 8), which are encoded as the spatial modes of photons. C_*l*_ are the corresponding amplitudes. For the initial Hamiltonian *H*_0_, the space is expanded by the basis of 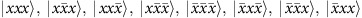. The polarization of single photons is rotated using HWPs and quarter-wave plate (QWPs), and the spatial modes are separated by BDs, each with a beam displacement of either 3.0 mm (BD30) or 6.0 mm (BD60). The state preparation is illustrated in the pane labelled Pre. The basis rotations BR1, BR2 and BR3 are used to express the input states in terms of the eigenstates of *H*_1_, *H*_2_ and *H*_0_, respectively. The dissipative evolutions DE0, DE1 and DE2 drive the input states to the ground states of *H*_0_, *H*_1_ and *H*_2_, respectively. Some of the detailed basis representations of the spatial modes are given in the right column. The solid magenta rings represent the preserved optical modes and the dashed magenta rings represent the discarded optical modes. The states indicated near the optical modes represent the corresponding preserved basis in the eight-dimensional space. Two types of measurements are performed, that is, TM and FM. Beam splitters (BSs) are used to send the photons to different measurement instruments. *H*, *V*, *R* and *D* represent the horizontal polarization, vertical polarization, right-hand circular polarization and diagonal polarization, respectively. Finally, photons are detected using single-photon detectors (SPDs).

Figure 2 shows the experimental setup for the investigation of the MZMs (see Methods). Our quantum simulator is realized by single photons. The states of three spin-1/2 sites correspond to a 2^3^-dimensional Hilbert space, which are encoded in the optical spatial modes of single photons (the image of the eight optical modes can be found in Supplementary Note 7). The spatial propagation of the photon in one direction is equivalent to the imaginary time evolution of (lower-dimensional) system. It is worth noting that during the mapping between the many-body-to-single-particle Hilbert spaces the notion of locality is lost: excitations that are nearby in the system, which is simulated, do not correspond to nearby spatial modes in the photonic system. Nevertheless, there are intriguing similarities. The basis of the spatial modes of photons is the same as that of the corresponding Hilbert space of three-spin system (see Supplementary Note 6 for more details).

The basis of a spin state can be expressed as the eigenstates of *σ*^*z*^ (denoted by |*z*〉, with an eigenvalue of 1, and 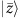, with an eigenvalue of −1), *σ*^*y*^ (denoted by |*y*〉, with an eigenvalue of 1, and 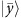 with an eigenvalue of −1) or *σ*^*x*^ (denoted by |*x*〉, with an eigenvalue of 1, and 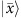, with an eigenvalue of −1). In our experiment, we employ beam displacers (BDs) of various lengths to prepare the initial states. A BD is a birefringent crystal, in which light beams with horizontal and vertical polarizations are separated by a certain displacement (depending on the length of the crystal)^37^. Two types of BDs are employed, BD30 (with a beam displacement of 3.0 mm) and BD60 (with a beam displacement of 6.0 mm). The polarization of the photon can be rotated using half-wave plates (HWPs) and the relative amplitudes of the different spatial modes can be conveniently adjusted. To obtain the ground states of the corresponding Hamiltonian, the polarization of the photons is used as the environmental degree of freedom. The coupling between the spatial mode and the polarization is achieved using HWPs, which rotate the polarization in the corresponding paths. Dissipative evolution is accomplished by passing the photons through a polarization beam splitter (PBS), which transmits the horizontal component and reflects the vertical component. In our case, only photons in the optical modes that have horizontal polarizations are preserved; these modes correspond to the ground states of the Hamiltonian. The components with vertical polarizations are completely dissipated. The precision of the dissipative evolution is dependent on the ratio between the reflected and transmitted parts of the vertical polarization after the PBS, which can be higher than 500:1. To ensure dissipative dynamics using a PBS, we must express the input states in the basis of the eigenstates of the corresponding Hamiltonian, which are referred to basis rotations. The eigenstates of 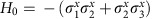 can be expressed as 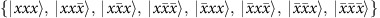, which are represented by the eight spatial modes of the single photon and are shown in the state preparation pane labelled by Pre in Fig. 2. The corresponding cross-sections of the spatial modes are shown in the right column in Fig. 2. Only the terms of |*xxx*〉 and 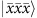 are preserved after the ITE operation of *H*_0_, which corresponds to two spatial modes in the dissipative evolution of DE0. For the ITE operation of 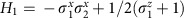, we should only consider the term of 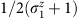 (the term 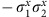 is the same as that in *H*_0_). Two HWPs with the angles setting to be 45° in the basis rotation BR1 are implemented in modes of |*xxx*〉 and 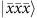, in which the basis of the first spin is rotated to |*z*〉 and 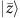. The two spatial modes are then separated to four by a BD60 with the spatial modes represented as 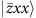, |*zxx*〉, 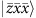 and 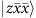. After the dissipative evolution of DE1, only the terms of 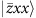 and 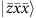 are preserved. The ITE operation of *H*_2_ can be implemented by the same way with the details given in Supplementary Note 6.

**Figure 3:**
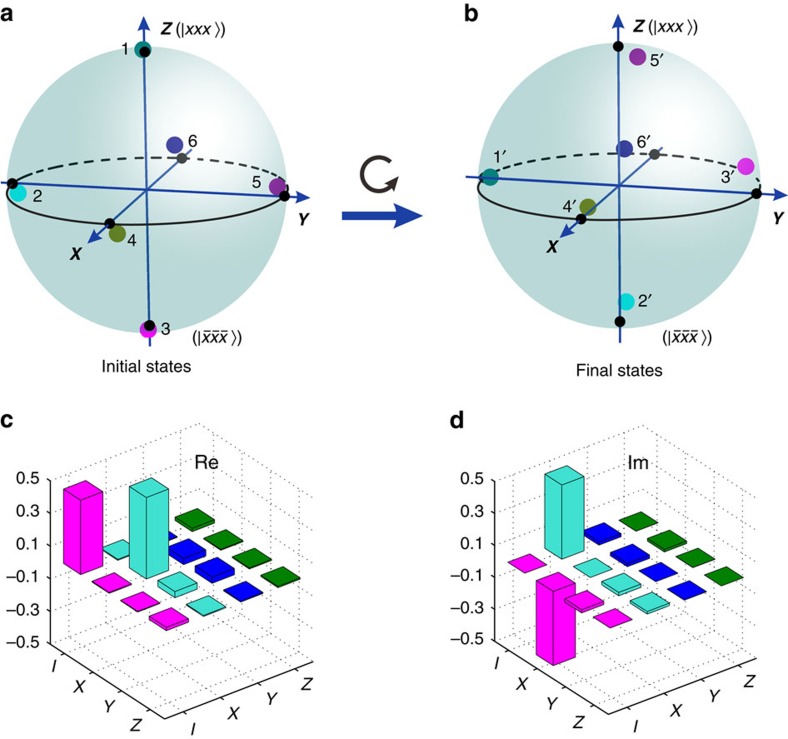
Experimental results on simulating the braiding evolution. (**a**) The six experimental initial states after the first dissipative evolution DE0 with the dark green dot labelled as 1, cyan dot labelled as 2, magenta dot labelled as 3, dark yellow dot labelled as 4, violet dot labelled as 5 and navy dot labelled as 6 in the Bloch sphere. (**b**) The corresponding experimental final states after the second DE0 with the dark green dot labelled as 1′, cyan dot labelled as 2′, magenta dot labelled as 3′, dark yellow dot labelled as 4′, violet dot labelled as 5′ and navy dot labelled as 6′ in the Bloch sphere. The black dots in the poles of the Bloch spheres represent the corresponding theoretical predictions with the states |*xxx*〉 (+***Z*** direction), 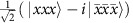 (−***Y*** direction), 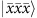 (−***Z*** direction), 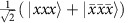 (+***X*** direction, |0_3s_〉), 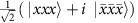 (+***Y*** direction) and 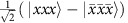 (−***X*** direction, |1_3s_〉), respectively. Owing to the experimental errors, the coloured dots (experimental results) are slightly separated from the corresponding black dots. The final states are shown to be rotated along the ***X*** axis by *π*/2 from the initial states. (**c**) Real (Re) and (**d**) imaginary (Im) parts of the exchange operator in the basis of {*I*(identity), *X*(*σ*^*x*^), *Y*(*σ*^*y*^), *Z*(*σ*^*z*^)}, with a fidelity of 94.13±0.04%.

To clearly show the two-mode output states, the corresponding density matrices, *ρ*, are expressed in the basis of {*I*,*σ*^*x*^,*σ*^*y*^,*σ*^*z*^} as follows: 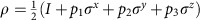. Here *I* represents the identity operator and *p*_1_, *p*_2_ and *p*_3_ are the corresponding real amplitudes, which uniquely identify density matrices on a Bloch sphere. The initial states after the ITE operation of DE0 is shown in Fig. 3a. The corresponding final states after the operator *U*_ex_ are illustrated in Fig. 3b. It is shown that the final state is the same as the initial state when the initial state is |0_3s_〉 (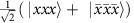, denoted as dot 4) or |1_3s_〉 (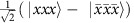, denoted as dot 6), which suggests that there is no off-diagonal elements in the braiding matrix, *U*_ex_. In addition, the relative geometric phase between |0_3s_〉 and |1_3s_〉 during the exchanging can be directly measured. Indeed, the final states are obtained by rotating the initial states counterclockwise along the **X** axis through an angle of *π*/2 when the initial state is a superposition state of |0_3s_〉 and |1_3s_〉, that is, the basis |0_3s_〉 obtains an extra phase factor of −*i* relative to |1_3s_〉. Thus, the braiding matrix can be determined by the relative geometric phase giving *U*_ex_=diag(1,*e*^*iπ*/2^). The braiding matrix determined here agrees well with the theoretical result^21^. This implies that any input state 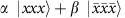, with the complex coefficients of *α* and *β* (|*α*|^2^+|*β*|^2^=1), would be transformed to 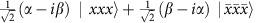, that is, the state 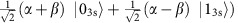 would be changed to 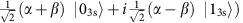 in the basis of |0_3s_〉 and |1_3s_〉.

The real and imaginary parts of the experimentally determined operator of the exchange process (the computation basis are represented as |*xxx*〉 and 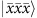), as determined via the quantum process tomography (represented by Pauli operators {*I*,*X*(*σ*^*x*^),*Y*(*σ*^*y*^), *Z*(*σ*^*z*^)})^38^, are presented in Fig. 3c,d. The fidelity is calculated to be 94.13±0.04% (the errors are deduced from the Poissonian photon counting noise and the detailed method is shown in Supplementary Note 8).

If the statistics between the MZMs were Abelian then the braiding matrix between these two degenerate states would have been the identity matrix without any relative phase between the degenerate basis states. The fact that we obtain a relative phase factor is in agreement with the non-Abelian character of MZMs.

### Experimental results on simulating local noises immunity

**Figure 4:**
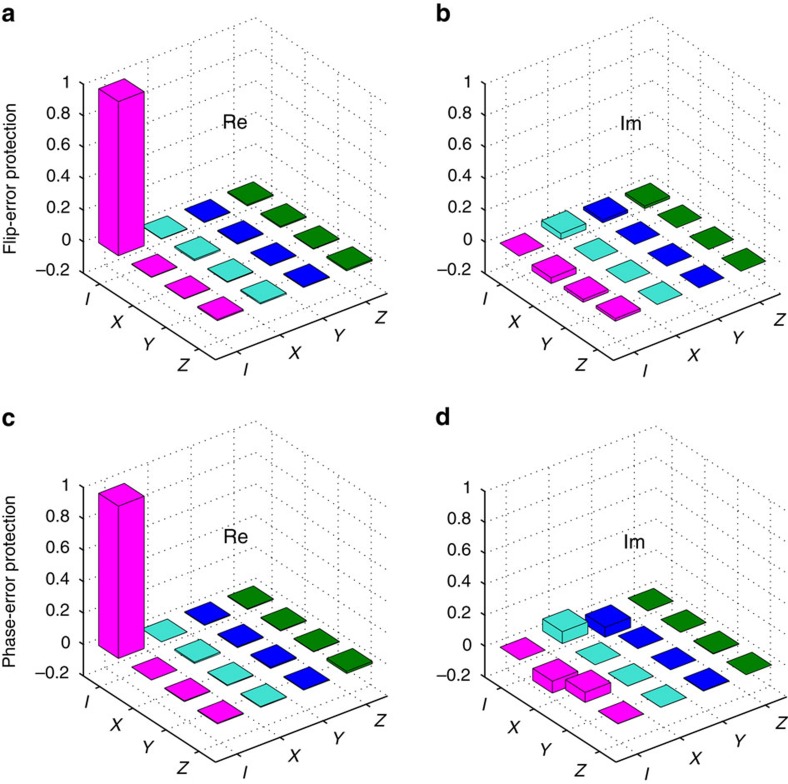
Experimental results on simulating local noises immunity. (**a**) Real (Re) and (**b**) imaginary (Im) parts of the flip-error protection operator, with a fidelity of 97.91±0.03%. (**c**). Real (Re) and (**d**) imaginary (Im) parts of the phase-error protection operator, with a fidelity of 96.99±0.04%. The basis are expressed as {*I*(identity), *X*(*σ*^*x*^), *Y*(*σ*^*y*^), *Z*(*σ*^*z*^)}.

We further investigate local noises immunity of the information encoded in the ground space of the KCM. The output state after the dissipative evolution DE0, 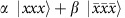, is treated as the initial state. After the operation of 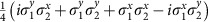 (flip-error operator), the two output modes would become eight. The final state is the same as the initial one by projecting the state back to the ground space of *H*_0_ (that is, the operation of DE0 and we omit the unobservable overall phase). Similarly, the initial two output modes (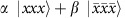) become four modes after the local phase noise operation of 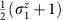. The final state is projected back to the initial one with the ITE operation of 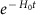 (see Supplementary Note 6 for more details). In our experiment, the system is subjected to a projection back onto the initial state after applying the errors. By doing so, the success probability is small, as the preserved state is just a fraction of the total state. However, it serves well for establishing the technology and methodology for the in-principle demonstration of the anyonic physics. Figure 4a,b show the real and imaginary parts of the flip-error protection operator with a fidelity of 97.91±0.03%. Figure 4c,d show the real and imaginary parts of the phase-error protection operator with a fidelity of 96.99±0.04%. The errors of the fidelity values are deduced from the Poissonian photon counting noise. This fact reveals the protection from the local flip error and phase error in the KCM. The total operation behaves as an identity, thus demonstrating immunity against noise. More experimental results can be found in Supplementary Note 9.

In general, the fidelity of protection operators are also affected by the noises without Z_2_ symmetry and the evolution time in the ITE. Owing to the fact that the environment can be well controlled in our optical simulator, the setup established here can also be used to investigate the effect of the noise without Z_2_ symmetry in the ground space of the TFIM. Our simulator offers the possibility to study the effect of other types of noise, which can be presented in the real system supporting MZMs (superconductor-based system). A central advantage of the optical simulation is that all the implementations can be realized with high precision.

## Discussion

In this study we experimentally investigated the physics of MZMs emerging in the KCM by simulating it with an equivalent photonic system. The photonic quantum simulator, which employs a set of projective measurements, represents a general and powerful approach to directly study quantum evolutions. We were able to transport two MZMs at the endpoints of a chain and measure the geometric phases resulting from their exchange. Moreover, we experimentally demonstrated that the degenerate subspace of the MZMs is resilient against local perturbations.

Our simulation is based on a non-local JW transformation that maps the fermionic KCM to an equivalent spin model. This transformation, although it changes the states of the system, does not alter the associated unitary evolutions, thus allowing us to measure directly the braiding matrix of MZMs. The appeal of our photonic quantum simulator is based on the fact that it allows for the transport of MZMs. It offers the exciting future possibility to investigate the braiding character of muti-MZM systems and demonstrate their non-Abelian statistics.

Applications towards topological quantum computation may be possible by employing the versatile and well-controlled optical quantum simulator presented here. MZMs and their braiding cannot be used to perform universal quantum computations^15^. However, one can envision implementing specifically tailored quantum algorithms, such as the Deutch–Josza algorithm^39^ where the information will be topologically protected during the whole process.

Owing to the fact that we encode the multi-site spin-1/2 system in the spatial modes of a single photon, the scalability of this method is limited. Nevertheless, it serves well for demonstrating the fundamental statistical properties and robustness of MZMs with such a small system size. Our main focus is on the physics and establishing the technology and the methodology. The gained knowhow (investigating the braiding of anyonic quasiparticles through the ITE) can be picked up by other technologies that offer scalability, such as the trapped ions^13^ or Josephson junctions^14^ system.

## Methods

### The ITE operator on states

For a given Hamiltonian *H* with a complete set of eigenstates |*e*_*k*_〉 and the corresponding eigenvalues *E*_*k*_, any arbitrary pure state |*φ*〉 can be expressed as


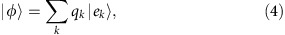


with *q*_*k*_ representing the corresponding complex amplitude. Here we focus on pure states, but the argument is also valid for mixed states. The corresponding ITE operator (*U*)^28^ on the state becomes


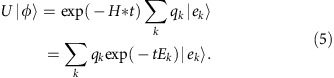


The evolution time *t* is chosen to be 5 in our analysis, which is long enough to drive the input state to the ground state of *H*. After the ITE, the amplitude *q*_*k*_ is changed to be *q*_*k*_exp(−5*E*_*k*_). We can see that the decay of the amplitude is strongly (exponentially) dependent on the energy: the higher the energy, the faster the decay of the amplitude. Therefore, only the ground states (with lowest energy) survives during the evolution. Furthermore, due to the fact that the Hamiltonian *H*_0_, *H*_1_ and *H*_2_ can be divided into two commuting parts, we can separate each ITE operator to two ITE operators whose eigenvectors can be directly obtained (see equation (3)).

### Experimental process in probing braiding characteristics

The experimental setup for probing braiding characteristics is shown in Fig. 2. The process follows the logical diagram presented in the pane enclosed in the black solid line, denoted by Logi. The setup used to prepare the input state is illustrated in the pane labelled Pre. For simplicity, at the beginning of the evolution, all three qubits are expressed in the basis of *σ*^*x*^, in which the ground states of *H*_0_ are encoded in the two spatial modes |*xxx*〉 and 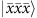. The dissipative evolution of *H*_0_ with arbitrary input states is illustrated in the pane labelled DE0, the output state of which is treated as the initial state. The basis rotation BR1 is employed and the subsequent dissipative evolution (DE1) should drive the state to approximate the ground state of *H*_1_.

After BR2 and DE2, the two input modes are expanded to four output modes, which are then sent to BR3, to implement the dissipative evolution DE0. In our experiment, we perform two types of measurements, that is, a two-mode measurement (TM) and a four-mode measurement (FM). The initial states, which contain spatial information, are transformed into states that contain polarization information, on which standard quantum-state tomography of polarization states can be performed. The TM corresponds to the tomography of the polarization states of a single qubit, for which four types of polarization measurements are required: horizontal polarization (*H*), vertical polarization (*V*), right-hand circular polarization (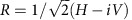) and diagonal polarization (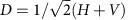). The polarization-analysis setup is constructed using a quarter-wave plate, a HWP and a PBS. The photon is then detected using a single-photon detector. Similarly, the FM corresponds to the measurement of 2-qubit polarization states and 16 measurements are required. In the TM and FM, beam splitters are used to send photons to different measurement instruments. The preparation of the initial states for the demonstration of noise immunity is the same as is shown in Pre and DE0. The disturbance operation is then implemented. After a second DE0, a TM is used to reconstruct the final states (see Supplementary Notes 10 and 11 for more details).

### Data availability

The data that support the findings of this study are available from the authors on request.

## Additional information

**How to cite this article:** Xu, J.-S. *et al.* Simulating the exchange of Majorana zero modes with a photonic system. *Nat. Commun.* **7,** 13194 doi: 10.1038/ncomms13194 (2016).

## Supplementary Material

Supplementary Information Supplementary Figures 1-13, Supplementary Table 1, Supplementary Notes 1-11 and Supplementary References.
