# Supplementary material for: Simulating the exchange of Majorana zero modes with a photonic system
Source: Nat Commun. 2016 Oct 25;7:13194. doi: 10.1038/ncomms13194 (PMC5093330; doi:10.1038/ncomms13194)
Supplement: Supplementary Information — Supplementary Figures 1-13, Supplementary Table 1, Supplementary Notes 1-11 and Supplementary References. [file ncomms13194-s1.pdf]

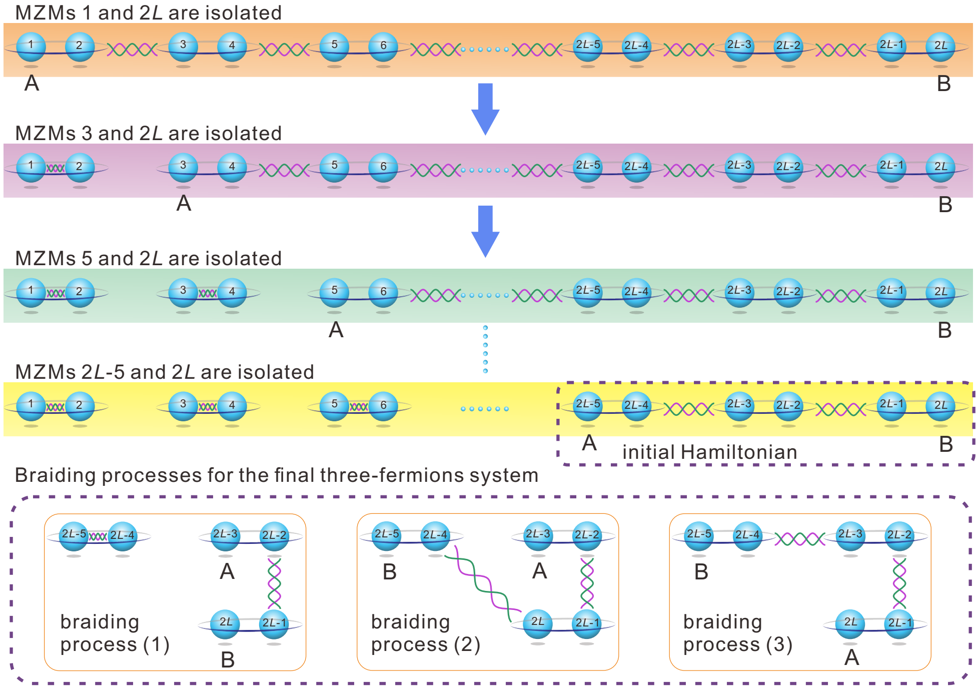

Supplementary Figure 1: The braiding process for L-fermions in the Kitaev Chain model (KCM). It can be reduced to the process for the final three-fermions, which is represented by the dark blue dashed panes. The spheres with numbers at their centers represent the Majorana fermions at the corresponding sites. A pair of Majorana fermions bounded by an enclosing ring represents a normal fermion. The wavy lines between different sites represent the interactions between them.

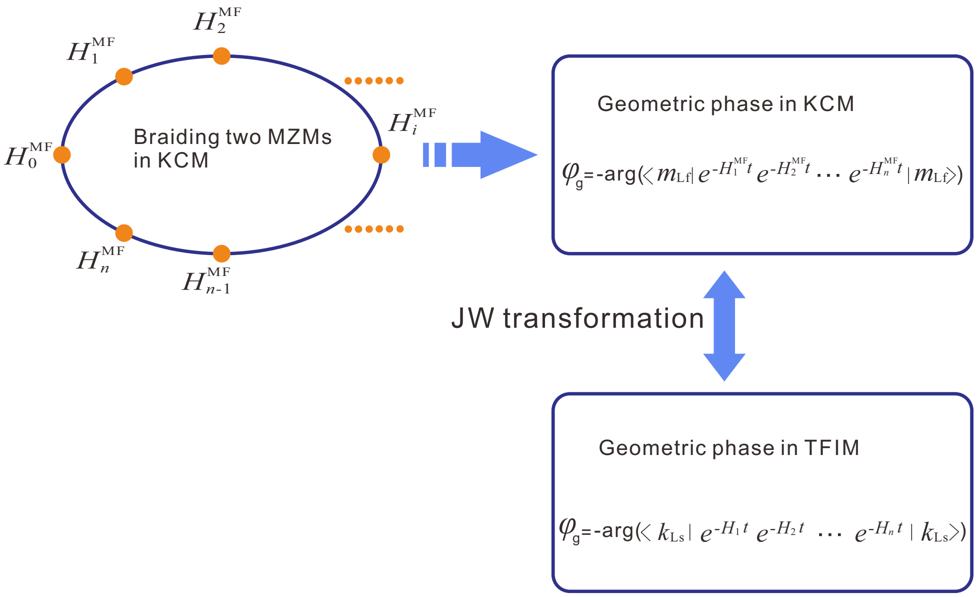

Supplementary Figure 2: The mapping of the braiding process. In the Kitaev Chain model (KCM), the braiding is determined by a set of Hamiltonian, H_0^{MF}, H_1^{MF}, $\cdots$, H_n^{MF}, $H_{n+1}^{\mathrm{MF}} = H_0^{\mathrm{MF}}$. The geometric phases obtained during the process can be uniquely determined by these Hamiltonians through Bargmann invariants. Under the Jordan-Wigner (JW) transformation, the geometric phases can be determined in the transverse-field Ising model (TFIM). The states $|m_{\mathrm{Lf}}\rangle$ and $|k_{\mathrm{Ls}}\rangle$ are a basis of the ground-state space defined in the KCM and in the TFIM (m and k=0 or 1), respectively.

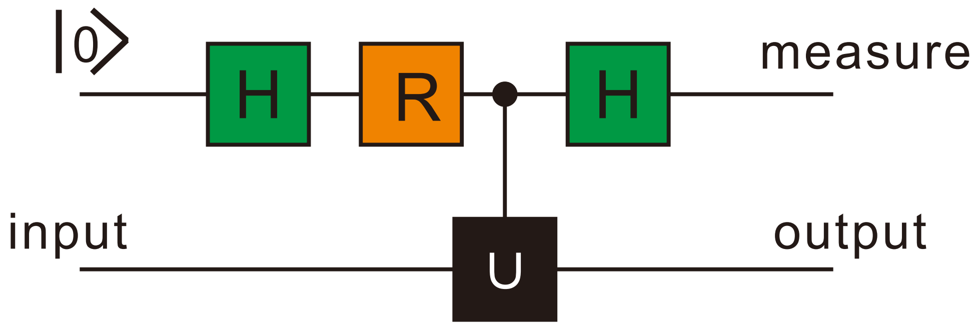

Supplementary Figure 3: The circuit of one step cooling algorithm. H represents the Hadamard gate operation. R represents a local phase gate and U is the unitary operation which is dependent on the Hamiltonian H_{s}.

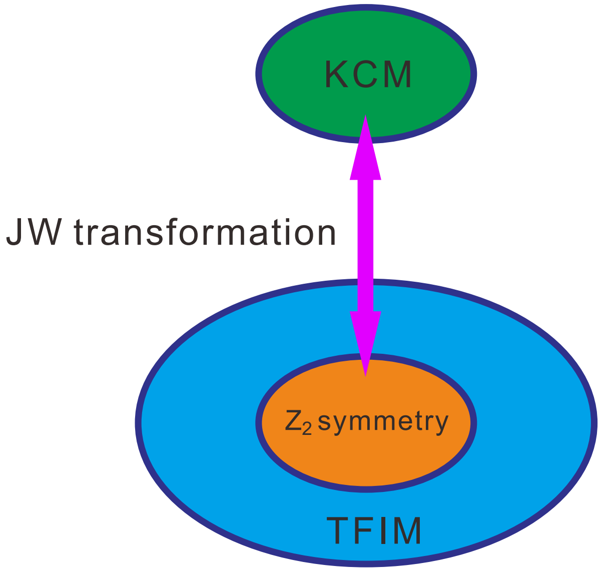

Supplementary Figure 4: The one-to-one mapping between different models. The mapping can be constructed between Kitaev Chain model (KCM) and the Z_2 symmetry subspace of transverse-field Ising model (TFIM) through the Jordan-Wigner (JW) transformation. In the Z_2 symmetry subspace, the degeneracy of the ground state of TFIM is protected. However, the degeneracy can be lifted by noises that do not respect the Z_2 symmetry.

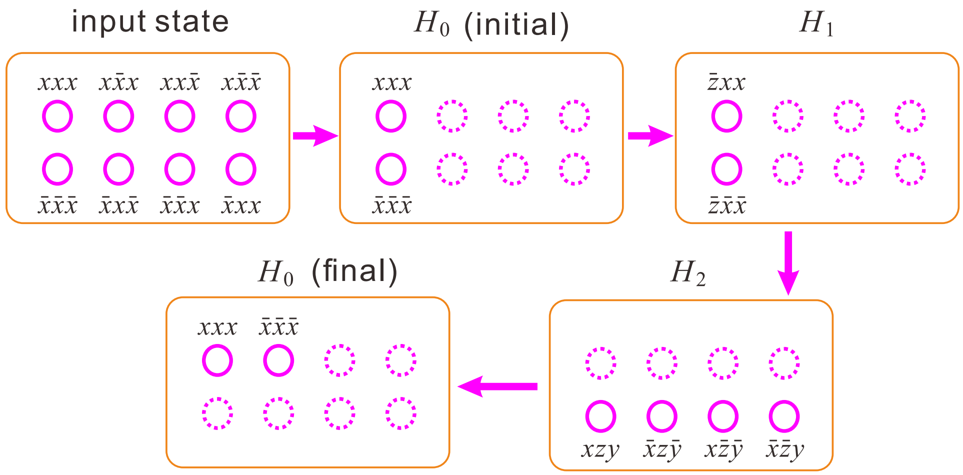

Supplementary Figure 5: Spatial modes of the output states for simulating the exchange of Majorana zero modes. The solid magenta rings represent the preserved optical modes, and the dashed magenta rings represent the discarded optical modes. The states indicated near the optical modes represent the corresponding preserved basis in the eight-dimensional space.

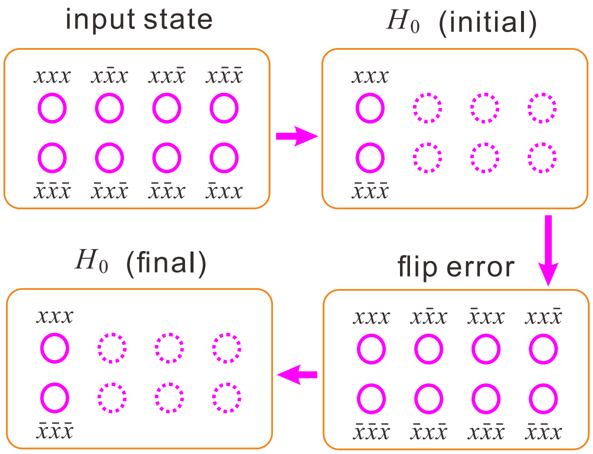

Supplementary Figure 6: Spatial modes of the output states for the investigation of the flip-error immunity. The solid magenta rings represent the preserved optical modes, and the dashed magenta rings represent the discarded optical modes. The states indicated near the optical modes represent the corresponding preserved basis in the eight-dimensional space.

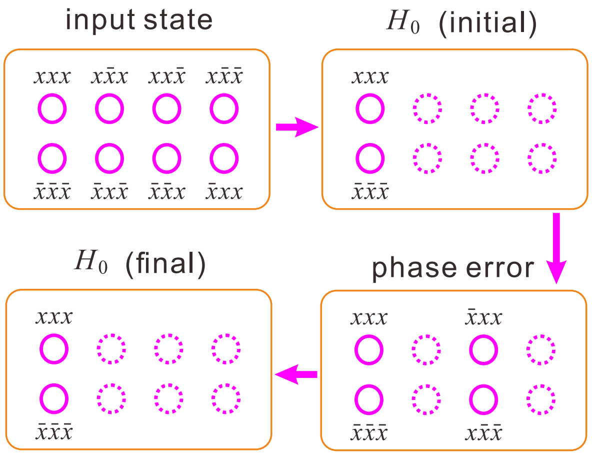

Supplementary Figure 7: Spatial modes of the output states for the investigation of the phase-error immunity. The solid magenta rings represent the preserved optical modes, and the dashed magenta rings represent the discarded optical modes. The states indicated near the optical modes represent the corresponding preserved basis in the eight-dimensional space.

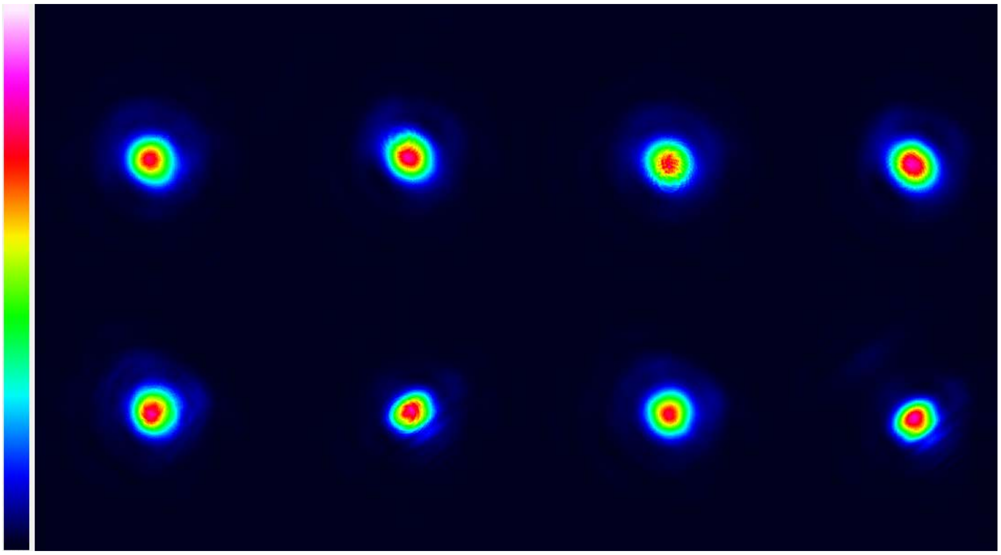

Supplementary Figure 8: Experimental image of the eight optical modes.

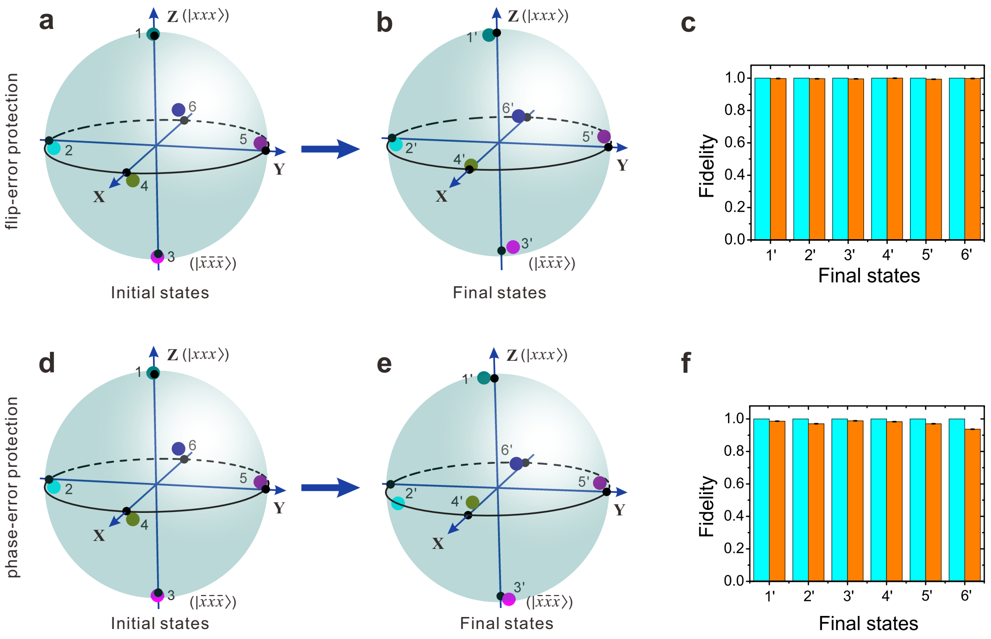

Supplementary Figure 9: Experimental results on simulating local noises immunity. **a.** and **d.** The six experimental initial states (after the first dissipative evolution DE0) with the dark green dots labeled as 1, cyan dots labeled as 2, magenta dots labeled as 3, dark yellow dots labeled as 4, violet dots labeled as 5 and navy dots labeled as 6 in the Bloch spheres. **b.** and **e.** The corresponding experimental final states (after the second dissipative evolution DE0) with the dark green dots labeled as $1'$, cyan dots labeled $2'$, magenta dots labeled as $3'$, dark yellow dots labeled as $4'$, violet dots labeled as $5'$, and navy dots labeled as $6'$ in the Bloch spheres for the cases of flip-error protection and phase-error protection, respectively. The black dots in the poles of the Bloch spheres represent the corresponding theoretical predictions with the states $|xxx\rangle$ (+**Z** direction), $\frac{1}{\sqrt{2}}(|xxx\rangle - i|\bar{x}\bar{x}\bar{x}\rangle)$ (-**Y** direction), $|\bar{x}\bar{x}\bar{x}\rangle$ (-**Z** direction), $\frac{1}{\sqrt{2}}(|xxx\rangle + |\bar{x}\bar{x}\bar{x}\rangle)$ (+**X** direction, $|0_{3s}\rangle$), $\frac{1}{\sqrt{2}}(|xxx\rangle + i|\bar{x}\bar{x}\bar{x}\rangle)$ (+**Y** direction), and $\frac{1}{\sqrt{2}}(|xxx\rangle - |\bar{x}\bar{x}\bar{x}\rangle)$ (-**X** direction, $|1_{3s}\rangle$), respectively. Due to the experimental errors, the coloured dots (experimental results) are slightly separated from the corresponding black dots. **c.** and **f.** the comparison between the fidelities of the final states (dark yellow columns) and the theoretical prediction (identity, cyan columns) for the cases of flip-error protection and phase-error protection, respectively.

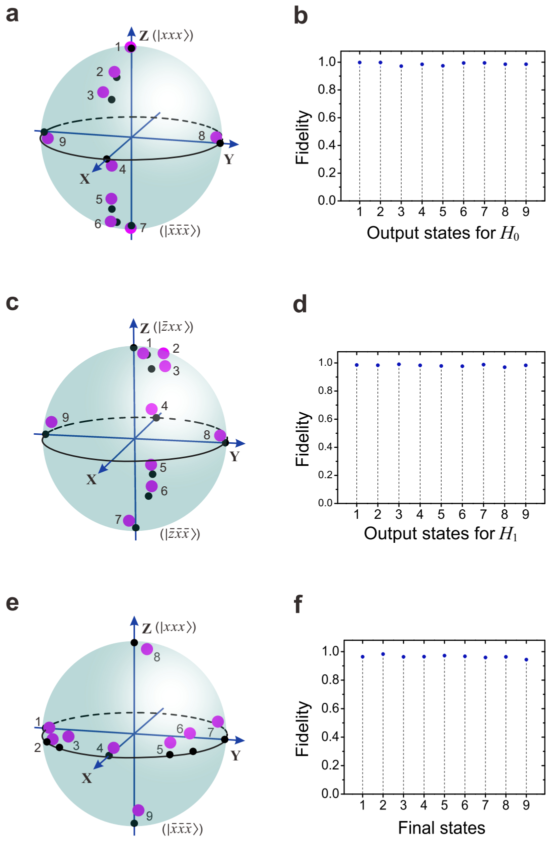

Supplementary Figure 10: More experimental results. **a.** Theoretical predictions (black dots) and experimental results (magenta dots) for the chosen nigh-ground states of H_0 (after the first dissipative evolution DE0) in a Bloch sphere. The corresponding theoretical states (corresponding numbers from 1 to 9) are represented as $|xxx\rangle$, $\frac{3}{\sqrt{10}}|xxx\rangle + \frac{1}{\sqrt{10}}|\bar{x}\bar{x}\bar{x}\rangle$, $\frac{2}{\sqrt{5}}|xxx\rangle + \frac{1}{\sqrt{5}}|\bar{x}\bar{x}\bar{x}\rangle$, $\frac{1}{\sqrt{2}}(|xxx\rangle + |\bar{x}\bar{x}\bar{x}\rangle)$, $\frac{1}{\sqrt{5}}|xxx\rangle + \frac{2}{\sqrt{5}}|\bar{x}\bar{x}\bar{x}\rangle$, $\frac{1}{\sqrt{10}}|xxx\rangle + \frac{3}{\sqrt{10}}|\bar{x}\bar{x}\bar{x}\rangle$, $|\bar{x}\bar{x}\bar{x}\rangle$, $\frac{1}{\sqrt{2}}(|xxx\rangle + i|\bar{x}\bar{x}\bar{x}\rangle)$, and $\frac{1}{\sqrt{2}}(|xxx\rangle - i|\bar{x}\bar{x}\bar{x}\rangle)$, respectively. **b.** The corresponding fidelities of the states in **a**. **c.** Theoretical predictions (black dots) and experimental results (magenta dots) for the nigh-ground states of H_1 (after dissipative evolution DE1) in a Bloch sphere. The corresponding theoretical states (corresponding numbers from 1 to 9) are represented as $|\bar{z}xx\rangle$, $\frac{3}{\sqrt{10}}|\bar{z}xx\rangle - \frac{1}{\sqrt{10}}|\bar{z}\bar{x}\bar{x}\rangle$, $\frac{2}{\sqrt{5}}|\bar{z}xx\rangle - \frac{1}{\sqrt{5}}|\bar{z}\bar{x}\bar{x}\rangle$, $\frac{1}{\sqrt{2}}(|\bar{z}xx\rangle - |\bar{z}\bar{x}\bar{x}\rangle)$, $\frac{1}{\sqrt{5}}|\bar{z}xx\rangle - \frac{2}{\sqrt{5}}|\bar{z}\bar{x}\bar{x}\rangle$, $\frac{1}{\sqrt{10}}|\bar{z}xx\rangle - \frac{3}{\sqrt{10}}|\bar{z}\bar{x}\bar{x}\rangle$, $|\bar{z}\bar{x}\bar{x}\rangle$, $\frac{1}{\sqrt{2}}(|\bar{z}xx\rangle + i|\bar{z}\bar{x}\bar{x}\rangle)$, and $\frac{1}{\sqrt{2}}(|\bar{z}xx\rangle - i|\bar{z}\bar{x}\bar{x}\rangle)$, respectively. **d.** The corresponding fidelities of the states in **c**. **e.** Theoretical predictions (black dots) and experimental results (magenta dots) for the final output states in a Bloch sphere for the investigation of non-trivial statistics (after the second dissipative evolution DE0). The theoretical states in **e** are obtained by rotating the corresponding states in **a** counterclockwise along the **X** axis through an angle of $\pi/2$. **f.** The corresponding fidelities of the states in **e**. Due to the experimental errors, the magenta dots (experimental results) are slightly separated from the corresponding black dots. The error bars for fidelities, which correspond to the counting statistics, are smaller than the size of the symbols.

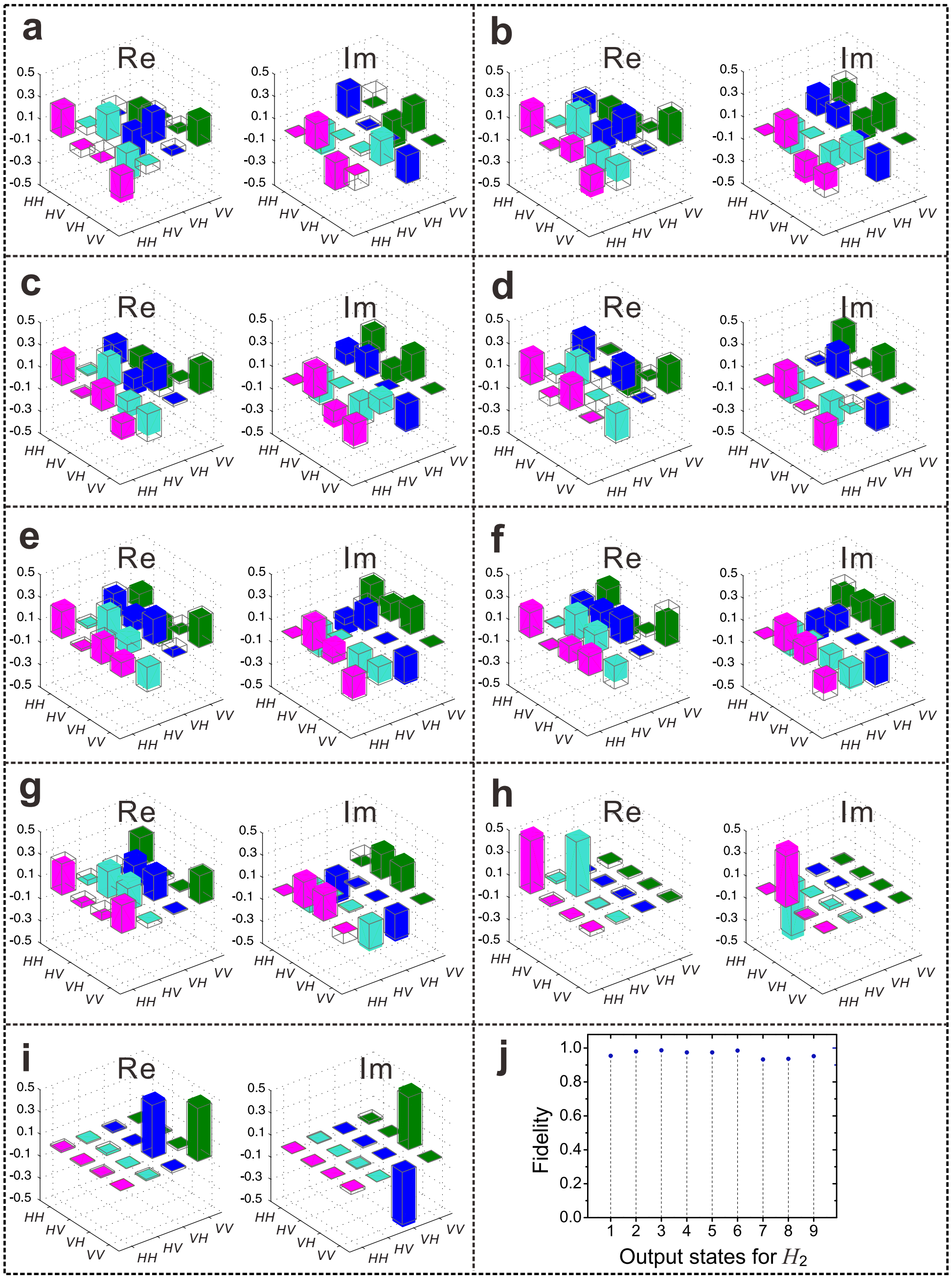

Supplementary Figure 11: The theoretical (solid columns) and experimental (open columns) density matrices of the output states after dissipative evolution DE2. The corresponding initial states after the first dissipative evolution DE0 are **a.** $|xxx\rangle$, **b.** $\frac{3}{\sqrt{10}}|xxx\rangle + \frac{1}{\sqrt{10}}|\bar{x}\bar{x}\bar{x}\rangle$ **c.** $\frac{2}{\sqrt{5}}|xxx\rangle + \frac{1}{\sqrt{5}}|\bar{x}\bar{x}\bar{x}\rangle$, **d.** $\frac{1}{\sqrt{2}}(|xxx\rangle + |\bar{x}\bar{x}\bar{x}\rangle)$, **e.** $\frac{1}{\sqrt{5}}|xxx\rangle + \frac{2}{\sqrt{5}}|\bar{x}\bar{x}\bar{x}\rangle$, **f.** $\frac{1}{\sqrt{10}}|xxx\rangle + \frac{3}{\sqrt{10}}|\bar{x}\bar{x}\bar{x}\rangle$, **g.** $|\bar{x}\bar{x}\bar{x}\rangle$, **h.** $\frac{1}{\sqrt{2}}(|xxx\rangle + i|\bar{x}\bar{x}\bar{x}\rangle)$ and **i.** $\frac{1}{\sqrt{2}}(|xxx\rangle - i|\bar{x}\bar{x}\bar{x}\rangle)$. The left- and right-hand columns in each pane represent the real (Re) and imaginary (Im) parts of the corresponding density matrices. **j.** The fidelities of the four-mode output states. The numbers from 1 to 9 represent the cases from **a** to **i**.

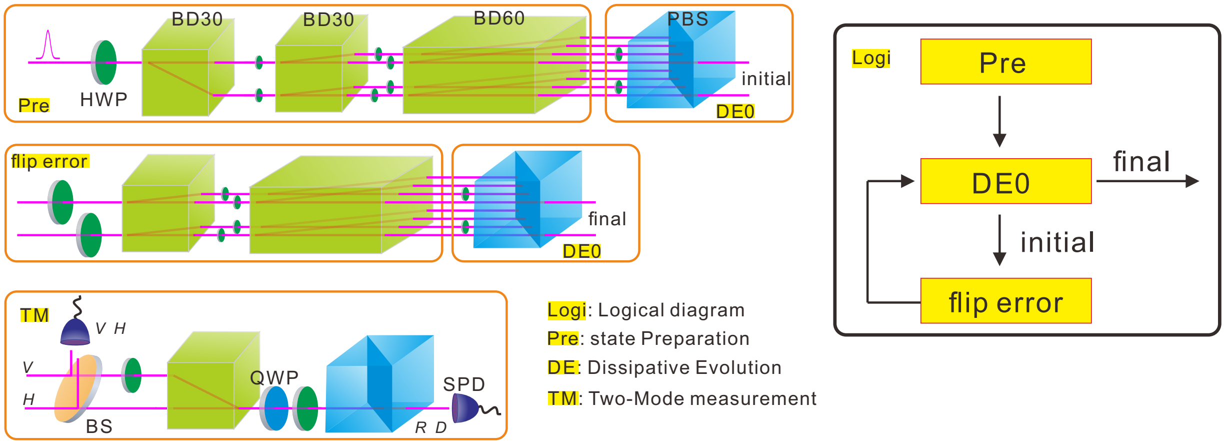

Supplementary Figure 12: Experimental setup for the investigation of the flip-error immunity. The process follows the logical diagram provided in the pane enclosed by the black solid line, denoted by Logi. The polarization of photons is rotated using half-wave plates (HWPs), and the spatial modes are separated by beam displacers, each with a beam displacement of either 3.0 mm (BD30) or 6.0 mm (BD60). The state preparation is illustrated in the pane labeled Pre. The dissipative evolution DE0 is illustrated in the pane that contains HWPs and a polarization beam splitter (PBS), the output states of which are treated as the initial states. The flip-error operation is achieved by six HWPs, a BD30 and a BD60. Two of the eight output modes are preserved after the second DE0. The initial and final states are measured in the two-mode measurement (TM) setup, in which the spatial information contained in the states is transformed into polarization information. Four polarization measurements are required: horizontal polarization (H), vertical polarization (V), right-hand circular polarization (R) and diagonal polarization (D). A beam splitter (BS) is used to send the photons to different measurement instruments. A quarter-wave plate (QWP), a HWP and a PBS are used for polarization analysis. Finally, photons are detected by single-photon detectors (SPDs).

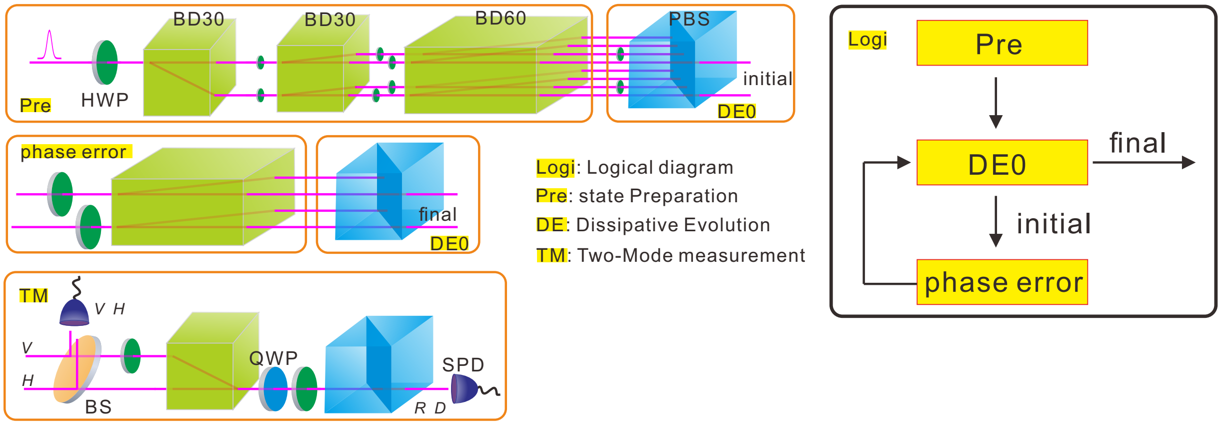

Supplementary Figure 13: Experimental setup for the investigation of the phase-error immunity. The process follows the logical diagram provided in the pane enclosed by the black solid line, denoted by Logi. The polarization of photons is rotated using half-wave plates (HWPs), and the spatial modes are separated by beam displacers, each with a beam displacement of either 3.0 mm (BD30) or 6.0 mm (BD60). The state preparation is illustrated in the pane labeled Pre. The dissipative evolution DE0 is illustrated in the pane that contains HWPs and a polarization beam splitter (PBS), the output states of which are treated as the initial states. The phase-error operation is achieved by two HWPs and a BD60. Two of the four output modes, with the first particle rotated by σ_z, are discarded after the second DE0. The other output modes are preserved. The initial and final states are measured in the two-mode measurement (TM) setup, in which the spatial information contained in the states is transformed into polarization information. Four polarization measurements are required: horizontal polarization (H), vertical polarization (V), right-hand circular polarization (R) and diagonal polarization (D). A beam splitter (BS) is used to send the photons to different measurement instruments. A quarter-wave plate (QWP), a HWP and a PBS are used for polarization analysis. Finally, photons are detected by single-photon detectors (SPDs).

Supplementary Table 1: Comparison between the Kitaev chain and the spin chain

**Models**	**Kitaev Chain Model**	**Transverse Field Ising Model**
Hamiltonian	$i(J\sum_{i=1}^{L-1}\gamma_{2i}\gamma_{2i+1}+\mu\sum_{i=1}^{L}\gamma_{2i-1}\gamma_{2i})$	$J\sum_{i=1}^{L-1}\sigma_i^x\sigma_{i+1}^x+\frac{\mu}{2}\sum_{i=1}^{L}\sigma_i^z$
Symmetry	Z_2 (degeneracy when $J>\mu\geq 0$)	Z_2 (degeneracy in the ferromagnetic region)
Degeneracy	Topological protected	Can be lifted by operators without Z_2 symmetry
Topological order	Yes	No
Basis in the ground space of a 3-particle system	$\|1_{3f}\rangle=\frac{1}{2}(\|000\rangle+\|011\rangle+\|101\rangle+\|011\rangle)$<br>$\|0_{3f}\rangle=\frac{1}{2}(\|001\rangle+\|010\rangle+\|100\rangle+\|111\rangle)$	$\|1_{3s}\rangle=\frac{1}{2}(\|\downarrow\downarrow\downarrow\rangle+\|\downarrow\uparrow\uparrow\rangle+\|\uparrow\downarrow\uparrow\rangle+\|\uparrow\uparrow\downarrow\rangle)$<br>$\|0_{3s}\rangle=\frac{1}{2}(\|\downarrow\downarrow\uparrow\rangle+\|\downarrow\uparrow\downarrow\rangle+\|\uparrow\downarrow\downarrow\rangle+\|\uparrow\uparrow\uparrow\rangle)$
Prepared initial states	Fixed fermion parity ($\|0_{3f}\rangle$ or $\|1_{3f}\rangle$)	Any superposition of $\|0_{3s}\rangle$ and $\|1_{3s}\rangle$
Flip error	$c_i^\dagger c_{i+1}$	$\frac{1}{4}(i\sigma_i^y\sigma_{i+1}^x+\sigma_i^y\sigma_{i+1}^y+\sigma_i^x\sigma_{i+1}^x-i\sigma_i^x\sigma_{i+1}^y)$
Phase error	$c_i^\dagger c_i$	$(\sigma_i^z+1)/2$

SUPPLEMENTARY NOTE 1. THE EXCHANGE OF MAJORANA ZERO MODES IN THE KITAEV CHAIN MODEL

The typical Hamiltonian in the KCM [1] reads as follows: $H = \omega \sum_{j=1}^{L}(c_j - c_j^\dagger)(c_{j+1} + c_{j+1}^\dagger)$, where ω represents an experimental parameter and c_j ($c_j^\dagger$) represents the fermionic annihilation (creation) operator. If we introduce the Majorana operators, $\gamma_{2j-1} = c_j^\dagger + c_j$ and $\gamma_{2j} = i(c_j^\dagger - c_j)$, the Hamiltonian becomes $H = i\omega \sum_{j=1}^{L-1} \gamma_{2j}\gamma_{2j+1}$ (ω is set to 1 in the main text). It can be seen that the Majorana fermions γ_{2j} and γ_{2j+1} are paired by interactions, but γ_1 and γ_{2L}, which correspond to two Majoranas, remain free. The basis of the two-fold-degenerate ground-state space of this system can be defined as the eigenstates of the operator $\tilde{c}_f^\dagger \tilde{c}_f$ ($\tilde{c}_f = \frac{1}{2}(\gamma_1 + i\gamma_{2L})$), explicitly, $|0_{\mathrm{Lf}}\rangle = \frac{1}{2^{(L-1)/2}} \sum_{p=0}^{\lfloor (L-1)/2 \rfloor} \sum_{i_1<i_2<\cdots<i_{2p+1}}^{L} c_{i_{2p+1}}^\dagger \cdots c_{i_1}^\dagger |vac\rangle$ (for the superposition of states with odd-fermion parity) and $|1_{\mathrm{Lf}}\rangle = \frac{1}{2^{(L-1)/2}}[1 + \sum_{p=1}^{\lfloor L/2 \rfloor} \sum_{i_1<i_2<\cdots<i_{2p}}^{L} c_{i_{2p}}^\dagger \cdots c_{i_1}^\dagger]|vac\rangle$ (for the superposition of states with even-fermion parity). The Z_2 symmetry and the ground-state degeneracy of this system are topologically protected. The braiding of the MZMs in a L-site KCM can be completed by a set of adiabatic processes, as shown in the Supplementary Figure 1.

For a general L-site KCM model with MZMs (A and B), at the sites 1 and $2L$, we can easily drive the isolated Majorana mode A from site 1 to the site $2K-1$ ($K < L$ determined by the minimal fermions system to demonstrate the braiding, here $K = L-2$) by employing a set of such adiabatic process:

$$H_{\mathrm{ad}}(\lambda) = i\Big[\sum_{j=1}^{m-1} \frac{1}{2}\gamma_{2j-1}\gamma_{2j} + \sum_{j=m+1}^{L} \gamma_{2j}\gamma_{2j+1} + (1-\lambda)\gamma_{2m}\gamma_{2m+1} + \frac{\lambda}{2}\gamma_{2m-1}\gamma_{2m}\Big]. \tag{1}$$

The parameter λ adiabatically changes from 0 to 1, the Hamiltonian will change from

$$H_m = i\Big(\sum_{j=1}^{m-1} \frac{1}{2}\gamma_{2j-1}\gamma_{2j} + \sum_{j=m}^{L} \gamma_{2j}\gamma_{2j+1}\Big), \tag{2}$$

with Majorana mode A located at the site $2m-1$ to the Hamiltonian

$$H_{m+1} = i\Big(\sum_{j=1}^{m} \frac{1}{2}\gamma_{2j-1}\gamma_{2j} + \sum_{j=m+1}^{L} \gamma_{2j}\gamma_{2j+1}\Big), \tag{3}$$

with Majorana mode A located at the site $2m+1$. As a result, the Majorana mode A moves

a step from left to right. With K such processes, Majorana mode A moves from the left boundary to the site $2K-1$.

As shown in Supplementary Figure 1, the box in the right denotes a small system to demonstrate the braiding. A four-fermion system has been proposed with a superconducting wire [2]. After that, more theoretical schemes for an implementation or simulation of the Kitaev model are proposed [3–5]. Here, without considering a concrete physical realization, we theoretically proposed that the three-fermion system is also possible to complete the braiding (details of the braiding processes in the box will be introduced in Supplementary Note 5).

When the braiding process in the box is finished, Majorana mode B will be located at $2K-1$ and Majorana mode A is located at $2L$. Finally, Majorana mode B at site $2K-1$ can be driven back to site 1 to complete the entire braiding process by a set of the inverse adiabatic evolutions introduced before.

It has been shown that the braiding transformations will introduce different geometric phases in the basis of the ground-space of the initial system. Measuring the geometric phases in this basis is a key part in demonstrating the statistical property of the MZMs [6]. Here, we propose to measure the geometric phases by a set of projective measurements $P_1, P_2, \cdots, P_n$ [7]. The projective measurement P_i is uniquely determined by the Hamiltonian H_i as $e^{-H_i t}$ with a large time t. The operator $e^{-H_i t}$ preserves the fermion parity of the initial state which guarantees the adiabatic condition during the braiding. The projective measurements can be realized by an imaginary time evolution algorithm, such as, the cooling algorithm in [8].

Unfortunately, in the one-chain fermionic system, the braiding effect (geometric phase as an overall phase) in this process can not be directly measured, because of the fixed fermion parity. However, through the Jordan-Wigner (JW) transformation, a general KCM can be transformed into a 1D transverse-field Ising model (TFIM) [9, 10] whose ground states can be prepared in any superposition state and the relative geometric phases can be finally measured. The method to probe the geometric phases in the KCM is presented in Supplementary Figure 2.

SUPPLEMENTARY NOTE 2. PRINCIPLE OF THE IMAGINARY TIME EVOLUTION

Here we explain in detail the basic idea of the imaginary time evolution (ITE) algorithm based on the cooling algorithm [8]. Each ITE operator can be realized by this method. A ITE operator can be successfully realized by a set of basic steps with some probability which is dependent on the character of the Hamiltonian and the initial state. Fortunately, in the braiding process for the KCM, the ITE operator can be efficiently realized.

The circuit for one step of the imaginary time evolution is shown in Supplementary Figure 3. The key components of the quantum circuit consists of four gates [8]: (a) two Hadamard gates

$$\mathsf{H} \equiv \frac{1}{\sqrt{2}}\left(|0\rangle + |1\rangle\right)\langle 0| + \frac{1}{\sqrt{2}}\left(|0\rangle - |1\rangle\right)\langle 1| \ , \tag{4}$$

applying to the ancilla qubit ($|0\rangle$ and $|1\rangle$ are the corresponding two levels) at the beginning and at the end of the quantum circuit; (b) a local phase gate,

$$\mathsf{R}\left(\alpha\right) \equiv |0\rangle\langle 0| - ie^{i\alpha}|1\rangle\langle 1| \ , \tag{5}$$

where the parameter α is chosen to optimize the efficiency of the algorithm; (c) a two-qubit controlled unitary operation,

$$\mathbb{1}\otimes|0\rangle\langle 0| + \mathsf{U}\otimes|1\rangle\langle 1| \ , \tag{6}$$

where $\mathbb{1}$ is the identity operator, and

$$\mathsf{U}\left(t\right) = e^{-iH_{\mathrm{s}}t} \tag{7}$$

is the real time evolution operator for the system. H_{s} is the Hamiltonian of the considered system (L-site KCM here), and t is the time of evolution which is another parameter we can use to optimize the efficiency of the algorithm. For a many-body Hamiltonian, we can well approximate the unitary evolution operator $\mathsf{U}(t)$ by the product of a set of local unitary operators through the Trotter-Suzuki expansion [11].

For any given initial state of the system, $|\psi_{\mathrm{in}}\rangle = \sum_{k=1}^{N_s}\sqrt{p_k}\sum_{l=1}^{n_k}\beta_{k,l}|e_{k,l}\rangle$, where N_{s} is the number of the eigenvector subspace of Hamiltonian H_{s}, n_k ($\sum_{k=1}^{N_s} n_k = 2^L$) is the degeneracy of the k-th eigenvector subspace of the Hamiltonian H_{s} with eigenvalue E_k, and $|e_{k,l}\rangle$ is the l-th eigenvector in this subspace. The probability to find the state in the k-th eigenvector

subspace is denoted by p_k. For each k, $\sum_{l=1}^{n_k} \beta_{k,l} |e_{k,l}\rangle$ is normalized, i.e. $\sum_{l=1}^{n_k} |\beta_{k,l}|^2 = 1$. Generally, we do not need to know the exact form of $|e_{k,l}\rangle$ and the phase between different eigenvector subspaces are not important in the algorithm.

The quantum circuit then produces the following output state:

$$\frac{1}{2}\sum_{k=1}^{N_s}\sqrt{p_k}(1 - ie^{-i(E_k t-\alpha)})\sum_{l=1}^{n_k}\beta_{k,l}|e_{k,l}\rangle\,|0\rangle + \frac{1}{2}\sum_{k=1}^{N_s}\sqrt{p_k}(1 + ie^{-i(E_k t-\alpha)})\sum_{l=1}^{n_k}\beta_{k,l}|e_{k,l}\rangle\,|1\rangle\,. \quad (8)$$

A measurement on the ancilla qubit in the computational basis $\{|0\rangle, |1\rangle\}$ yields the states

$$A_0\sum_{k=1}^{N_s}\sqrt{p_k}(1 - ie^{-i(E_k t-\alpha)})\sum_{l=1}^{n_k}\beta_{k,l}|e_{k,l}\rangle, \quad (9)$$

and

$$A_1\sum_{k=1}^{N_s}\sqrt{p_k}(1 + ie^{-i(E_k t-\alpha)})\sum_{l=1}^{n_k}\beta_{k,l}|e_{k,l}\rangle, \quad (10)$$

respectively, where A_0 (A_1) is the normalization factor. It is clear that the coefficient of the eigenvector subspace is modified by the factor $(1 - ie^{-i(E_k t-\alpha)})$ or $(1 + ie^{-i(E_k t-\alpha)})$ depending on the results of the ancilla qubit. As a result, the weight of the eigenvector subspace, especially the weight of the ground-state subspace, will be modified. To clarify this point, let us consider the module of the factor $(1 \pm ie^{-i(E_k t-\alpha)})$,

$$|(1 \pm ie^{-i(E_k t-\alpha)})|^2 = 2\left[1 \pm \sin(E_k t - \alpha)\right]. \quad (11)$$

If the parameters α and t are properly chosen, such that,

$$-\pi/2 \leq E_k t - \alpha \leq \pi/2, \quad (12)$$

for all the eigenvalue E_k, the function $\sin(E_k t - \alpha)$ will increase with the energy. Therefore, $1-\sin(E_k t-\alpha)$ $(1+\sin(E_k t-\alpha))$ will decrease (increase) with the increase of the eigenvalue. As a result, the weight of ground state of the system will increase along with the measurement result $|0\rangle$ on the ancilla qubit, and the weight of the ground state will decrease along with the the measurement result $|1\rangle$ on the ancilla qubit.

To make the parameters satisfy the condition introduced before, we normalize the Hamiltonian of the system to make the eigenvalues of the Hamiltonian in the range $[-1, 1]$. The general Hamiltonian in our braiding processes of MZMs in the L-site KCM can be easily normalized as

$$H' = i[\sum_{j=1}^{m}\frac{1}{2}\gamma_{2j-1}\gamma_{2j} + \sum_{j=m+1}^{L}\gamma_{2j}\gamma_{2j+1}]/L. \quad (13)$$

By setting $\alpha = 0$ and supposing the parameter t satisfies the condition: $E_k t \ll 1$, the weight of the eigenvectors of the Hamiltonian change as

$$1 - \sin(E_k t) \approx e^{-E_k t}, \tag{14}$$

which is the imaginary time evolution operator for small time t.

To realize a ITE with a large t, we divide it into k steps, where each step satisfies the condition introduced before. Thus, a total of k steps of ITE introduced before are applied on the initial state (we make no measurement during the cooling; we measure the qubits at the end of the manipulation). The final state we obtain is

$$|\psi_{\text{fin}}\rangle = \sum_{j=0}^{k} \sqrt{C_k^j} \Big(\sum_{i=1}^{N_s} \sqrt{p_i (1/2 + a_i)^{k-j} (1/2 - a_i)^j} \sum_{l=1}^{n_i} \beta_{i,l} |e_{i,l}\rangle |\varphi_{ji}\rangle \Big), \tag{15}$$

where $|\varphi_{ji}\rangle$ is a properly normalized state of the ancilla. $a_i = \frac{1}{2}\sin(E_i t - \alpha)$ and $\alpha = 0$. After the manipulations, we make measurement on the ancilla. The probability to get j $|0\rangle$ (the number of the success cooling manipulation) is,

$$C_k^j \sum_{i=1}^{2^n} p_i (1/2 + a_i)^{k-j} (1/2 - a_i)^j. \tag{16}$$

This is a mixture of several binomial distributions with the first one corresponding to the ground state. For a standard binomial distribution: $C_k^j p^j (1-p)^{k-j}$, the expected value is kp and the variance is $np(1-p)$. Thus, the concentration interval of the binomial distribution is between $k((\frac{1}{2} - a_i) - \frac{1}{\sqrt{k}}\sqrt{\frac{1}{4} - a_i^2})$ and $k((\frac{1}{2} - a_i) + \frac{1}{\sqrt{k}}\sqrt{\frac{1}{4} - a_i^2})$. In order to prepare the ground state of the system, the intersection between different binomial distribution should be very small. In other words

$$k((\frac{1}{2} - a_1) - \frac{1}{\sqrt{k}}\sqrt{\frac{1}{4} - a_1^2}) \gg k((\frac{1}{2} - a_2) + \frac{1}{\sqrt{k}}\sqrt{\frac{1}{4} - a_2^2}), \tag{17}$$

which is equivalent to

$$\sqrt{k}(a_2 - a_1) \gg \frac{1}{2} - (a_1^2 + a_2^2). \tag{18}$$

It should be noted that the other binomial distribution corresponding to higher energy have much less intersection with the distribution corresponding to ground states. Under the condition $E_k t \ll 1$ $(k = 1, 2, \cdots, 2^L)$, we have

$$\frac{1}{2} - (a_1^2 + a_2^2) \approx \frac{1}{2}, a_2 - a_1 \approx \frac{1}{2}\Delta t, \tag{19}$$

and the intersection condition can be simplify to

$$\sqrt{k}\Delta t \gg 1, \tag{20}$$

where Δ is the gap of the system. If this condition is satisfied, the binomial distribution of the ground state is sufficiently separated from the others, and the number of $|0\rangle$ outcomes during the measurement on the ancilla will be concentrated at $k(1-\frac{1}{2}a_1)$ with probability p_1. Thus, the number of measurements that successfully obtain the ground state of the system scale as

$$\frac{1}{p_1(\Delta t)^2}. \tag{21}$$

The gap of the Hamiltonian during the braiding decreases polynomially with L. In addition, the overlap between the ground state of $H_i^{\rm MF}$ and $H_{i+1}^{\rm MF}$ is independent on L. Therefore, the ITE operator $e^{-H_i t}$ with large t is polynomial scaled with L in the KCM Braiding situation.

SUPPLEMENTARY NOTE 3. THE RELATION BETWEEN KITAEV CHAIN MODEL AND TRANSVERSE-FIELD ISING MODEL

The Jordan-Wigner transformation gives a one-to-one mapping between the Kitaev chain model (KCM) and a subspace of the transverse-field Ising model (TFIM) which consists of all the operators with Z_2 symmetry. As mentioned in the main text, the geometric phase due to the braiding in the KCM can be directly mapped into this subspace. In addition, the information encoded on the ground space of the TFIM is robust against the local noise in this subspace.

The Z_2-invariant Ising model has two-degenerate ground states. There are two zero modes which are topologically protected by the boundary condition [9, 10]. As an example, the Ising model $J\sum_{i=1}^{L-1}\sigma_i^x\sigma_{i+1}^x$ without Z direction magnetic field has two basis in its ground state space: $|\rightarrow\rightarrow\cdots\rightarrow\rangle$ and $|\leftarrow\leftarrow\cdots\leftarrow\rangle$. If a local small Z direction magnetic field is introduced, the local flip of the basis of σ_x is occurred. These two basis can be connected in the following manner: $|\rightarrow\rightarrow\cdots\rightarrow\rangle \dashrightarrow |\rightarrow\cdots\rightarrow\leftarrow\rangle \dashrightarrow |\rightarrow\cdots\rightarrow\leftarrow\leftarrow\rangle \dashrightarrow \cdots \dashrightarrow |\rightarrow\rightarrow\leftarrow\cdots\leftarrow\rangle \dashrightarrow |\rightarrow\leftarrow\cdots\leftarrow\leftarrow\rangle \dashrightarrow |\leftarrow\leftarrow\cdots\leftarrow\rangle$. Thus the transformation between these two basis states can be induced and the tunneling rate is about $e^{-L/\zeta}$ where L is the length of the chain and ζ is the correlation length. When the length of the chain is very large, the degeneracy of the spin will be topologically protected.

Although the KCM and TFIM share the same mathematical structure they are governed by different physics. The major differences between them include: the initial state in the TFIM can be prepared in the superposition of $|0_{\mathrm{Ls}}\rangle$ and $|1_{\mathrm{Ls}}\rangle$; the degeneracy in the TFIM can be lifted by noises without Z_2 symmetry (such as $\sum_j \sigma_j^x$) and the excitations in the TFIM that corresponds to the MZMs in the KCM are non-local. The relation between KCM and TFIM is given schematically in Supplementary Figure 4. We further summarize the similarities and differences between them in Supplementary Table 1.

With the well controlled spin model, the whole system can always be in the degenerate subspace. Generally, the local noise can induce the tunneling between the MZMs whose amplitude is exponentially decaying with the distance. In our simulated spin system, the distance is very short. The tunneling between the MZMs will be induced if all the local noises are present. Fortunately, the noises in the optical system can be well controlled and the tunneling can be well suppressed by reducing some local noises. The robustness of the information encoded in the degenerate subspace against single local noises have been demonstrated in our experiment.

SUPPLEMENTARY NOTE 4. BARGMANN INVARIANTS AND THE GEOMETRIC PHASE

Generally, the angle (φ_{B}) obtained from the Bargmann invariant [12] associated with the three states ρ_1, ρ_2 and ρ_3 in the ray space is $\arg(tr(\rho_1\rho_2\rho_3))$ [13–15]. φ_{B} is gauge invariant and is equal to the negative geometric phase of φ_{g} which is associated with the geodesic triangle of the three states [13]. Actually, following the line of Pancharatnam [16], it can be extended to the situation with many states.

In KCM system, the geometric phase, associated with the basis $|i_{\mathrm{gf}}\rangle$ of the ground-state space of H_0^{MF}, obtained during the braiding can be determined by [7]

$$\begin{aligned} \varphi_{\mathrm{g0}} &= -\arg(\langle 0_{3\mathrm{f}}|e^{-H_2^{\mathrm{MF}}t}e^{-H_1^{\mathrm{MF}}t}|0_{3\mathrm{f}}\rangle) \quad , \\ \varphi_{\mathrm{g1}} &= -\arg(\langle 1_{3\mathrm{f}}|e^{-H_2^{\mathrm{MF}}t}e^{-H_1^{\mathrm{MF}}t}|1_{3\mathrm{f}}\rangle) \quad , \end{aligned} \tag{22}$$

where φ_{g0} (φ_{g1}) is the geometric phase of the basis $|0\rangle$ ($|1\rangle$) in the ground space of Hamiltonian H_0^{MF} and t is a large number, such as 5. The ITE operators $e^{-H_1^{\mathrm{MF}}t}$ and $e^{-H_2^{\mathrm{MF}}t}$ both preserve the fermion parity of the initial state which guarantees the adiabatic condition during the braiding. Introducing the Jordan-Wigner (JW) transformation

$U_{\rm JW}$ as: $U_{\rm JW}\gamma_1U_{\rm JW}^{-1}=\sigma_1^x$ $,U_{\rm JW}\gamma_2U_{\rm JW}^{-1}=-\sigma_1^y$ $,U_{\rm JW}\gamma_3U_{\rm JW}^{-1}=-\sigma_1^z\sigma_2^x$ $,U_{\rm JW}\gamma_4U_{\rm JW}^{-1}=\sigma_1^z\sigma_2^y$ $,U_{\rm JW}\gamma_5U_{\rm JW}^{-1}=\sigma_1^z\sigma_2^z\sigma_3^x$ $,U_{\rm JW}\gamma_6U_{\rm JW}^{-1}=-\sigma_1^z\sigma_2^z\sigma_3^y$ where γ_i represents the corresponding Majorana operator. The geometric phases become

$$\begin{aligned}\varphi_{\rm g0}&=-\arg(\langle 0_{\rm 3f}|U_{\rm JW}^{-1}U_{\rm JW}e^{-H_2^{\rm MF}t}U_{\rm JW}^{-1}U_{\rm JW}e^{-H_1^{\rm MF}t}U_{\rm JW}^{-1}U_{\rm JW}|0_{\rm 3f}\rangle)\quad,\\ \varphi_{\rm g1}&=-\arg(\langle 1_{\rm 3f}|U_{\rm JW}^{-1}U_{\rm JW}e^{-H_2^{\rm MF}t}U_{\rm JW}^{-1}U_{\rm JW}e^{-H_1^{\rm MF}t}U_{\rm JW}^{-1}U_{\rm JW}|1_{\rm 3f}\rangle)\quad.\end{aligned}\tag{23}$$

It can be expressed in the spin model as

$$\begin{aligned}\varphi_{\rm g0}&=-\arg(\langle 0_{\rm 3s}|e^{-H_2t}e^{-H_1t}|0_{\rm 3s}\rangle)\quad,\\ \varphi_{\rm g1}&=-\arg(\langle 1_{\rm 3s}|e^{-H_2t}e^{-H_1t}|1_{\rm 3s}\rangle)\quad,\end{aligned}\tag{24}$$

where $|0_{\rm 3s}\rangle$ ($|1_{\rm 3s}\rangle$) is the mapping of $|0_{\rm 3f}\rangle$ ($|1_{\rm 3f}\rangle$) under JW transformation, and it is the basis of the ground space of H_0. All the physical quantities have been transformed into a spin system.

To determine the geometric phases, we prepare the initial state in $|\phi_0\rangle=\alpha|0_{\rm 3s}\rangle+\beta|1_{\rm 3s}\rangle$ with α and β representing the complex numbers (not normalized). We then apply the operator $U_{\rm ex}=e^{-H_2t}e^{-H_1t}$ to this state. The final state is determined and the relative geometric phase are obtained. If we prepare the initial state as $|0_{\rm 3s}\rangle$ or $|1_{\rm 3s}\rangle$, the state after the operator $U_{\rm ex}$ is still the same which can be used to determine the off-diagonal elements of the braiding matrix [7]. In this experiment, we perform a tomography to measure the effect of the operator $U_{\rm ex}$ in the ground space of H_0, and the information of the braiding matrix is thus determined [7]. A benefit of this method is that we can also prepare the initial state by the imaginary evolution method which needs only the Hamiltonian H_0.

SUPPLEMENTARY NOTE 5. THE ADIABATIC EXCHANGE PROCESS IN THE SIMPLEST KITAEV CHAIN MODEL

As mentioned in the main text and above, the braiding of two MZMs can be completed in the three-fermion KCM [10]. Now, we explicitly illustrate the adiabatic braiding process in this system (see Figure 1 in the main text). Throughout the entire braiding process, the Hamiltonian can be described in the general form

$$H^{\rm F}=-t_{12}(c_1^+-c_1)(c_2^++c_2)-t_{23}(c_2^+-c_2)(c_3^++c_3)-it_{13}(c_1^+-c_1)(c_3^+-c_3)+\mu_1c_1^+c_1,\tag{25}$$

where c_i^+ and c_i represent the creation and annihilation fermionic operators at position i. t_{ij} represents the interaction amplification between the particles in positions i and j. μ_1 (≥ 0) represents the chemical potential at site 1.

The Majorana operators can be introduced as follows:

$$\begin{aligned}
\gamma_1 &= c_1 + c_1^+ \quad , \\
\gamma_2 &= i(c_1^+ - c_1) \quad , \\
\gamma_3 &= c_2 + c_2^+ \quad , \\
\gamma_4 &= i(c_2^+ - c_2) \quad , \\
\gamma_5 &= c_3 + c_3^+ \quad , \\
\gamma_6 &= i(c_3^+ - c_3) \quad .
\end{aligned} \tag{26}$$

The inverse relations between the Fermi operators and the Majorana operators are

$$\begin{aligned}
c_1 &= (\gamma_1 + i\gamma_2)/2 \quad , \\
c_1^+ &= (\gamma_1 - i\gamma_2)/2 \quad , \\
c_2 &= (\gamma_3 + i\gamma_4)/2 \quad , \\
c_2^+ &= (\gamma_3 - i\gamma_4)/2 \quad , \\
c_3 &= (\gamma_5 + i\gamma_6)/2 \quad , \\
c_3^+ &= (\gamma_5 - i\gamma_6)/2 \quad .
\end{aligned} \tag{27}$$

The general Hamiltonian can be rewritten in terms of Majorana operators as (overall constants are ignored)

$$H^{\mathrm{MF}} = i(t_{12}\gamma_2\gamma_3 + t_{23}\gamma_4\gamma_5 + t_{13}\gamma_2\gamma_6 + \frac{\mu_1}{2}\gamma_1\gamma_2). \tag{28}$$

The initial Hamiltonian is defined by setting $t_{13} = 0$, $t_{12} = t_{23} = 1$ and $\mu_1 = 0$ as follows:

$$H_0^{\mathrm{MF}} = i(\gamma_2\gamma_3 + \gamma_4\gamma_5) \quad . \tag{29}$$

The ground state of the Hamiltonian H_0^{MF} is two-fold degenerate. The basis denoted by $|0_{3\mathrm{f}}\rangle$ and $|1_{3\mathrm{f}}\rangle$ consist of the eigenstates of the operator $\tilde{c}_f^\dagger \tilde{c}_f$ ($\tilde{c}_f = \frac{1}{2}(\gamma_1 + i\gamma_6)$). Explicitly, $|0_{3\mathrm{f}}\rangle = \frac{1}{2}(c_1^\dagger + c_2^\dagger + c_3^\dagger + c_3^\dagger c_2^\dagger c_1^\dagger)|\mathrm{Vac}\rangle$ and $|1_{3\mathrm{f}}\rangle = \frac{1}{2}(1 + c_2^\dagger c_1^\dagger + c_3^\dagger c_1^\dagger + c_3^\dagger c_2^\dagger)|\mathrm{Vac}\rangle$ where $|\mathrm{Vac}\rangle$ denotes the vacuum of c_is [2, 17]. The Majorana zero modes (MZMs) γ_1 and γ_6 are located at the endpoints of the chain.

Now we will show that the two isolated MZMs denoted by A and B, located at sites 1 and 6, in the KCM can be driven to complete the braiding transformation. The process is accomplished by adiabatically tuning the parameters in equation (28), where different geometric phases will be added to the states $|0_{3\mathrm{f}}\rangle$ and $|1_{3\mathrm{f}}\rangle$. The entire braiding process can be divided into the following three components:

(1) Let the parameters in the Hamiltonians described by equations (28) be

$$\begin{aligned} \mu_1 &= \lambda \quad, \\ t_{12} &= 1-\lambda \quad, \\ t_{13} &= 0 \quad, \\ t_{23} &= 1 \quad. \end{aligned} \tag{30}$$

The Hamiltonian then becomes

$$H^{\mathrm{MF}} = i[(1-\lambda)\gamma_2\gamma_3 + \gamma_4\gamma_5 + \frac{\lambda}{2}\gamma_1\gamma_2]. \tag{31}$$

When the parameter λ adiabatically changes from 0 to 1, the Hamiltonian will be transformed from H_0^{MF} to

$$H_1^{\mathrm{MF}} = i[\gamma_4\gamma_5 + \frac{1}{2}\gamma_1\gamma_2]. \tag{32}$$

At the same time, the Majorana mode A will move from site 1 to site 3.

(2) Let

$$\begin{aligned} \mu_1 &= 1-\lambda \quad, \\ t_{23} &= 1 \quad, \\ t_{13} &= \lambda \quad, \\ t_{12} &= 0 \quad. \end{aligned} \tag{33}$$

The Hamiltonians described by equations (28) becomes

$$H^{\mathrm{MF}} = i[\gamma_4\gamma_5 + \lambda\gamma_2\gamma_6 + \frac{1-\lambda}{2}\gamma_1\gamma_2]. \tag{34}$$

When λ adiabatically changes from 0 to 1, the Hamiltonian will be transformed from H_1^{MF} to

$$H_2^{\mathrm{MF}} = i(\gamma_4\gamma_5 + \gamma_2\gamma_6). \tag{35}$$

The Majorana mode B, initially located at site 6, will be driven to site 1. This adiabatic process is the key element of the braiding.

(3) Let

$$\begin{aligned} \mu_1 &= 0 \quad , \\ t_{13} &= 1-\lambda \quad , \\ t_{23} &= 1 \quad , \\ t_{12} &= \lambda \quad . \end{aligned} \tag{36}$$

The Hamiltonians described by equations (28) becomes

$$H^{\rm MF} = i[\lambda\gamma_2\gamma_3 + \gamma_4\gamma_5 + (1-\lambda)\gamma_2\gamma_6]. \tag{37}$$

When λ adiabatically changes from 0 to 1, the Hamiltonian will be transformed from $H_2^{\rm MF}$ back to $H_0^{\rm MF}$, and the Majorana mode A will be driven from site 3 to site 6. The braiding of the MZMs A and B is thus completed. The basis of the ground-state space will develop various geometric phases, which correspond to the non-Abelian characteristics of MZMs [2, 17].

Currently, the direct implementation of braiding in fermionic systems remains a considerable challenge. In addition, the geometric phases obtained in one chain model can not be directly measured in fermionic system. By employing the Jordan-Wigner (JW) transformation, we can transform the fermionic Hamiltonian into a spin-1/2 system. In the three-fermionic KCM, the JW transformation can be written as follows:

$$\begin{aligned} c_1^{+} &= \sigma_1^{+}/2 \quad , \\ c_1 &= \sigma_1^{-}/2 \quad , \\ c_1^{+}c_1 &= \sigma_1^{z}/2 + 1/2 \quad , \\ c_2^{+} &= e^{i\pi(\sigma_1^z+1/2)}\sigma_2^{+}/2 = -\sigma_1^z\sigma_2^{+}/2 \quad , \\ c_2 &= e^{-i\pi(\sigma_1^z+1/2)}\sigma_2^{-}/2 = -\sigma_1^z\sigma_2^{-}/2 \quad , \\ c_2^{+}c_2 &= \sigma_2^{z}/2 + 1/2 \quad , \\ c_3^{+} &= e^{i\pi(\sigma_1^z/2+\sigma_2^z/2+1)}\sigma_3^{+}/2 = \sigma_1^z\sigma_2^z\sigma_3^{+}/2 \quad , \\ c_3 &= e^{-i\pi(\sigma_1^z/2+\sigma_2^z/2+1)}\sigma_3^{-}/2 = \sigma_1^z\sigma_2^z\sigma_3^{-}/2 \quad , \\ c_3^{+}c_3 &= \sigma_3^{z}/2 + 1/2 \quad , \end{aligned} \tag{38}$$

where σ_i^+ $(= \sigma_i^x + i\sigma_i^y)$ and σ_i^- $(= \sigma_i^x - i\sigma_i^y)$ represent the raising and lowering operators of the spin at site i. After the JW transformation, a general KCM will be transformed into

a transverse-field Ising model (TFIM). The ground-state degeneracy in the ferromagnetic phase of the TFIM correspond to that of MZMs at both ends of the chain [10]. However, because of the non-local nature of the JW transformation, the MZMs in the KCM correspond to nonlocal excitations in the spin model, that is,

$$\begin{aligned}
\gamma_1 &= \sigma_1^x \quad , \\
\gamma_2 &= -\sigma_1^y \quad , \\
\gamma_3 &= -\sigma_1^z \sigma_2^x \quad , \\
\gamma_4 &= \sigma_1^z \sigma_2^y \quad , \\
\gamma_5 &= \sigma_1^z \sigma_2^z \sigma_3^x \quad , \\
\gamma_6 &= -\sigma_1^z \sigma_2^z \sigma_3^y \quad .
\end{aligned} \tag{39}$$

The braiding of non-local excitations is not well defined. However, the geometric phases and the braiding matrix corresponding to the braiding of two MZMs in the KCM is invariant under the JW transformation, and it can be also determined in the spin model (see the next section). To determine the matrix, the Hamiltonians, corresponding to H_0^{MF}, H_1^{MF} and H_2^{MF}, become

$$\begin{aligned}
H_0 &= -(\sigma_1^x \sigma_2^x + \sigma_2^x \sigma_3^x) \quad , \\
H_1 &= -\sigma_2^x \sigma_3^x + \frac{1}{2}(\sigma_1^z + 1) \quad , \\
H_2 &= -(\sigma_2^x \sigma_3^x + \sigma_1^x \sigma_2^z \sigma_3^y) \quad .
\end{aligned} \tag{40}$$

It is noted that all the interactions in the KCM are local (three-body next-nearest-neighbor interaction at most). The ground state of the Hamiltonian H_0 is also two-fold degenerate and the basis in the degenerate space is denoted by $|0_{3\mathrm{s}}\rangle$ and $|1_{3\mathrm{s}}\rangle$. Explicitly, $|0_{3\mathrm{s}}\rangle = \frac{1}{2}(|\uparrow\downarrow\downarrow\rangle + |\downarrow\uparrow\downarrow\rangle + |\downarrow\downarrow\uparrow\rangle + |\uparrow\uparrow\uparrow\rangle)$ and $|1_{3\mathrm{s}}\rangle = \frac{1}{2}(|\downarrow\downarrow\downarrow\rangle + |\uparrow\uparrow\downarrow\rangle + |\uparrow\downarrow\uparrow\rangle + |\downarrow\uparrow\uparrow\rangle$), where $|\downarrow\rangle$ and $|\uparrow\rangle$ represent spin up and spin down, respectively.

SUPPLEMENTARY NOTE 6. THE IMAGINARY-TIME EVOLUTION FOR STATES ENCODED IN OPTICAL SPATIAL MODES

For a given Hamiltonian H with a complete set of eigenstates $|e_k\rangle$ and the corresponding eigenvalues E_k, any arbitrary pure state $|\phi\rangle$ can be expressed as

$$|\phi\rangle = \sum_k q_k |e_k\rangle, \tag{41}$$

with q_k representing the corresponding complex amplitude. Here, we focus on pure states, but the argument is also valid for mixed states. The corresponding imaginary-time evolution (ITE) operator (U) [18] on the state becomes

$$U|\phi\rangle = \exp(-H * t)\sum_k q_k|e_k\rangle = \exp(-H * 5)\sum_k q_k|e_k\rangle = \sum_k q_k \exp(-5E_k)|e_k\rangle. \tag{42}$$

The evolution time t is chosen to be 5, which is long enough to drive the input state to the ground state of H in our analysis. After the ITE, the amplitude q_k is changed to be $q_k \exp(-5E_k)$. We can see that the decay of the amplitude is strongly (exponentially) dependent on the energy: the higher energy, the faster the decay of the amplitude. Therefore, only the ground states (with lowest energy) survives this evolution. Furthermore, due to the fact that the Hamiltonian H_0, H_1 and H_2 can be diveded into two commuted parts, we can separate the ITE operator to two ITE operators whose eigenvectors are very easy to obtain (see equation (2) in the main text).

We then show the detailed processes to probe the geometric phases and braiding matrix, and demonstrate the local noises immunity of MZMs.

Geometric phases and braiding matrix

(1) In our experiment, the state information is encoded in the optical spatial modes. The basis of a two-level system can be expressed in the eigenstates of σ^z (denoted by $|z\rangle$, with an eigenvalue of 1, and $|\bar{z}\rangle$, with an eigenvalue of -1), σ^y (denoted by $|y\rangle$, with an eigenvalue of 1, and $|\bar{y}\rangle$, with an eigenvalue of -1) or σ^x (denoted by $|x\rangle$, with an eigenvalue of 1, and $|\bar{x}\rangle$, with an eigenvalue of -1). The eigenstates of $H_0(= -(\sigma_1^x\sigma_2^x + \sigma_2^x\sigma_3^x))$ can be expressed as $\{|xxx\rangle, |xx\bar{x}\rangle, |x\bar{x}x\rangle, |x\bar{x}\bar{x}\rangle, |\bar{x}xx\rangle, |\bar{x}x\bar{x}\rangle, |\bar{x}\bar{x}x\rangle, |\bar{x}\bar{x}\bar{x}\rangle\}$. For the term of $-\sigma_1^x\sigma_2^x$, the largest factor e^5 is obtained when the products of eigenvalues for particles 1 and 2 are equal to 1, according to the ITE (equation (42)). For the other cases the products of eigenvalues for particles 1 and 2 are equal to -1. Hence, the added amplitude factor becomes e^{-5}, which is

negligible compared to e^5 (for any state with nonzero ground state probability, we can always increase its amplitude to arbitrary high level by increasing the efficiency of the ITE, i.e., by increasing the evolution time from 5 to ∞). As a result, only the terms involving the basis of $\{|xxx\rangle, |xx\bar{x}\rangle, |\bar{x}\bar{x}x\rangle, |\bar{x}\bar{x}\bar{x}\rangle\}$ are preserved after the projection of $e^{-\sigma_1^x\sigma_2^x*5}$. Similarly, after the projector $e^{-\sigma_2^x\sigma_3^x*5}$, the largest factor e^5 is obtained when the products of eigenvalues for particles 2 and 3 are equal to 1 and the preserved state would ivolve in the basis of $\{|xxx\rangle, |\bar{x}\bar{x}\bar{x}\rangle\}$. As a result, the ITE for H_0 can be written as

$$\begin{aligned} |\phi_0\rangle = U_0|\phi\rangle &= \sum_k q_k \exp(-H_0 * 5)|e_k\rangle \\ &= \sum_k q_k \exp(\sigma_2^x\sigma_3^x * 5)\exp(\sigma_1^x\sigma_2^x * 5)|e_k\rangle \\ &\simeq \alpha|xxx\rangle + \beta|\bar{x}\bar{x}\bar{x}\rangle, \end{aligned} \tag{43}$$

which is normalized with complex coefficients α and β ($|\alpha|^2 + |\beta|^2 = 1$). For simplicity, we would omit below the normalization of the output states.

(2) $|\phi_0\rangle$ is then subjected to the follow ITE

$$U_1 = \exp(-H_1 * 5), \tag{44}$$

with $H_1 = -\sigma_2^x\sigma_3^x + \frac{1}{2}(\sigma_1^z + 1)$. The eigenstates of H_1 can be expressed as $\{|zxx\rangle, |zx\bar{x}\rangle, |z\bar{x}x\rangle, |z\bar{x}\bar{x}\rangle, |\bar{z}xx\rangle, |\bar{z}x\bar{x}\rangle, |\bar{z}\bar{x}x\rangle, |\bar{z}\bar{x}\bar{x}\rangle\}$. The largest amplitudes are obtained when the product of eigenvalues of $\sigma_2^x\sigma_3^x$ is 1 (for the projector of $e^{-\sigma_2^x\sigma_3^x*5}$) and the eigenvalue of σ_1^z is -1 (for the projector of $e^{-\frac{1}{2}(\sigma_1^z+1))*5}$), which refer to the terms of $|\bar{z}xx\rangle$ and $|\bar{z}\bar{x}\bar{x}\rangle$. The input state can be written as (basis rotation)

$$|\phi_0\rangle = \alpha|xxx\rangle + \beta|\bar{x}\bar{x}\bar{x}\rangle = \alpha|zxx\rangle + \alpha|\bar{z}xx\rangle + \beta|z\bar{x}\bar{x}\rangle - \beta|\bar{z}\bar{x}\bar{x}\rangle, \tag{45}$$

where the first particle is expressed in the basis $|z\rangle$ and $|\bar{z}\rangle$. After the ITE, the state becomes

$$|\phi_1\rangle = \alpha|\bar{z}xx\rangle - \beta|\bar{z}\bar{x}\bar{x}\rangle. \tag{46}$$

(3) The state is then subjected to the ITE of

$$U_2 = \exp(-H_2 * 5), \tag{47}$$

with $H_2 = -(\sigma_2^x\sigma_3^x + \sigma_1^x\sigma_2^z\sigma_3^y)$. We express the first particle back to the basis $|x\rangle$ and $|\bar{x}\rangle$. The input state becomes

$$|\phi_1\rangle = \alpha|\bar{z}xx\rangle - \beta|\bar{z}\bar{x}\bar{x}\rangle = \alpha|xxx\rangle - \alpha|\bar{x}xx\rangle - \beta|x\bar{x}\bar{x}\rangle + \beta|\bar{x}\bar{x}\bar{x}\rangle. \tag{48}$$

The state $|\phi_1\rangle$ remains unchanged after the ITE $\exp(-\sigma_2^x\sigma_3^x * 5)$, for all of the corresponding amplitudes are imposed with an equal factor of e^5. For the Hamiltonian $-\sigma_1^x\sigma_2^z\sigma_3^y$, the corresponding eigenstates are $\{|xz\bar{y}\rangle, |xzy\rangle, |x\bar{z}\bar{y}\rangle, |x\bar{z}y\rangle, |\bar{x}z\bar{y}\rangle, |\bar{x}\bar{z}\bar{y}\rangle, |\bar{x}\bar{z}y\rangle, |\bar{x}zy\rangle\}$. The input state of $|\phi_1\rangle$ expresses on such basis becomes

$$\begin{aligned}|\phi_1\rangle &= \alpha|xxx\rangle - \alpha|\bar{x}xx\rangle - \beta|x\bar{x}\bar{x}\rangle + \beta|\bar{x}\bar{x}\bar{x}\rangle \\ &= \alpha|x\rangle|z+\bar{z}\rangle[(1+i)|\bar{y}\rangle + (1-i)|y\rangle] \\ &- \alpha|\bar{x}\rangle|z+\bar{z}\rangle[(1+i)|\bar{y}\rangle + (1-i)|y\rangle] \\ &- \beta|x\rangle|z-\bar{z}\rangle[(1-i)|\bar{y}\rangle + (1+i)|y\rangle] \\ &+ \beta|\bar{x}\rangle|z-\bar{z}\rangle[(1-i)|\bar{y}\rangle + (1+i)|y\rangle].\end{aligned} \tag{49}$$

The ITE process preserves the terms with the product of eigenvalues of particles 1, 2 and 3 being 1. As a result, the final state becomes

$$\begin{aligned}|\phi_2\rangle = U_2|\phi_1\rangle \simeq\, &[\alpha(1-i) - \beta(1+i)]|xzy\rangle \\ &+ [\alpha(1+i) + \beta(1-i)]|x\bar{z}\bar{y}\rangle \\ &+ [-\alpha(1+i) + \beta(1-i)]|\bar{x}z\bar{y}\rangle \\ &+ [-\alpha(1-i) - \beta(1+i)]|\bar{x}\bar{z}y\rangle.\end{aligned} \tag{50}$$

(4) We now come to the final ITE, where the Hamiltonian turns back to H_0. We need to express the state $|\phi_2\rangle$ on the basis $\{|xxx\rangle, |xx\bar{x}\rangle, |x\bar{x}x\rangle, |x\bar{x}\bar{x}\rangle, |\bar{x}xx\rangle, |\bar{x}x\bar{x}\rangle, |\bar{x}\bar{x}x\rangle, |\bar{x}\bar{x}\bar{x}\rangle\}$ and keep only the terms $|xxx\rangle$ and $|\bar{x}\bar{x}\bar{x}\rangle$. The final state becomes

$$|\phi_3\rangle = U_0|\phi_2\rangle \simeq (\alpha - i\beta)|xxx\rangle + (\beta - i\alpha)|\bar{x}\bar{x}\bar{x}\rangle. \tag{51}$$

It is more clear if we express the initial state $|\phi_0\rangle$ and the final state $|\phi_3\rangle$ in the basis $|z\rangle$ and $|\bar{z}\rangle$. $|\phi_0\rangle = [\alpha+\beta, \alpha-\beta, \alpha-\beta, \alpha+\beta, \alpha-\beta, \alpha+\beta, \alpha+\beta, \alpha-\beta]$ and $|\phi_3\rangle = [-i(\alpha+\beta), \alpha-\beta, \alpha-\beta, -i(\alpha+\beta), \alpha-\beta, -i(\alpha+\beta), -i(\alpha+\beta), \alpha-\beta]$, which shows the non-Abelian character.

Robustness against perturbation operation of $\frac{1}{4}(i\sigma_1^y\sigma_2^x + \sigma_1^y\sigma_2^y + \sigma_1^x\sigma_2^x - i\sigma_1^x\sigma_2^y)$

(1) For any input state with nonzero amplitude on the ground states of H_0, when it is subjected to the ITE U_0, the output state is the ground state of H_0 with the form of $|\phi_0\rangle = \alpha|xxx\rangle + \beta|\bar{x}\bar{x}\bar{x}\rangle$.

(2) After the perturbation operation of $\frac{1}{4}(i\sigma_1^y\sigma_2^x+\sigma_1^y\sigma_2^y+\sigma_1^x\sigma_2^x-i\sigma_1^x\sigma_2^y)$, the state becomes

$$\begin{aligned}|\phi_0^{'}\rangle &= \frac{1}{4}(i\sigma_1^y\sigma_2^x+\sigma_1^y\sigma_2^y+\sigma_1^x\sigma_2^x-i\sigma_1^x\sigma_2^y)(\alpha|xxx\rangle+\beta|\bar{x}\bar{x}\bar{x}\rangle)\\ &= \frac{1}{4}[(\alpha|xxx\rangle+\beta|\bar{x}\bar{x}\bar{x}\rangle)-(\alpha|x\bar{x}x\rangle+\beta|\bar{x}x\bar{x}\rangle)+(\alpha|\bar{x}xx\rangle+\beta|x\bar{x}\bar{x}\rangle)-(\alpha|\bar{x}\bar{x}x\rangle+\beta|xx\bar{x}\rangle)].\end{aligned} \tag{52}$$

The simulation of the perturbation operation is realized by the rotation of the half-wave plates in the corresponding spatial modes.

(3) The same ITE U_0 is applied to the state $|\phi_0^{'}\rangle$, where only the terms $|xxx\rangle$ and $|\bar{x}\bar{x}\bar{x}\rangle$ are preserved. As a result, the final state becomes

$$|\phi_f\rangle = \alpha|xxx\rangle+\beta|\bar{x}\bar{x}\bar{x}\rangle, \tag{53}$$

which is the same as the initial state and the protection of local site flip is shown.

Robustness against perturbation operation of $(\sigma_z+1)/2$

(1) For any input state with nonzero amplitude of ground states of H_0, when it is subjected to the ITE U_0, the output state is the ground state of H_0 with the form of $|\phi_0\rangle = \alpha|xxx\rangle + \beta|\bar{x}\bar{x}\bar{x}\rangle$.

(2) The perturbation operation $\frac{1}{2}(\sigma_1^z+1)$ means that there is a probability of 0.5 to rotate the particle 1 along σ^z direction and a probability of 0.5 to do nothing. As a result, the state would becomes

$$|\phi_0^{'}\rangle = (\alpha|xxx\rangle+\beta|\bar{x}\bar{x}\bar{x}\rangle)_{\mathrm{np}}+(\alpha|\bar{x}xx\rangle+\beta|x\bar{x}\bar{x}\rangle)_{\mathrm{pe}}, \tag{54}$$

where the subscripts np and pe represent the cases without and with perturbation, respectively. The setup is similar to the case of transforming the basis of particle 1 from σ^x to σ^z, as expressed in equation (45). However, the four output modes are denoted by $\{|xxx\rangle, |\bar{x}\bar{x}\bar{x}\rangle, |\bar{x}xx\rangle, |x\bar{x}\bar{x}\rangle\}$ for the perturbation operation and $\{|zxx\rangle, |\bar{z}xx\rangle, |z\bar{x}\bar{x}\rangle, |\bar{z}\bar{x}\bar{x}\rangle\}$ for the basis rotation.

(3) The same ITE U_0 is applied to the state $|\phi_0^{'}\rangle$, where only the terms $|xxx\rangle$ and $|\bar{x}\bar{x}\bar{x}\rangle$ are preserved. As a result, the final state becomes

$$|\phi_{\mathrm{f}}\rangle = \alpha|xxx\rangle+\beta|\bar{x}\bar{x}\bar{x}\rangle, \tag{55}$$

which is the same as the initial state and the property of local phase-error immunity is shown.

To illustrate the encoding process more clearly, the cross sections of the output spatial modes of each process are presented in Supplementary Figure 5 (exchange of MZMs), Supplementary Figure 6 (immunity against flip error) and Supplementary Figure 7 (immunity against phase error). The solid magenta rings represent the preserved optical modes, and the dashed magenta rings represent the discarded optical modes. The states indicated near the optical modes represent the corresponding preserved basis in the eight-dimensional space. The mapping between the basis of initial states and the optical modes is chosen to make the measurement of the final states (interference between different spatial modes) more convenient.

SUPPLEMENTARY NOTE 7. EXPERIMENTAL IMAGE OF THE EIGHT OPTICAL MODES AND THE DETECTION PROCESS

Supplementary Figure 8 presents an experimental images of the eight optical modes of equal amplitude. The image was obtained as the output of the Pre setup using a laser with a central wavelength of 800 nm and a repetition rate of 76 MHz. To investigate the braiding transformation, the input photon pulses are attenuated and filtered by a single-mode fiber and a 3 nm interference filter. Two of the eight output modes are preserved and the photons are detected by single-photon detectors. The number of counts for each measurement in our experiment is approximately 100K *counts/s*. The errors on the measured quantities related to the counting statistics are therefore small. Beam displacers provide stable interference between different spatial modes [19]. To achieve high visibility of the interference between different spatial modes, the gradients of the beam displacers are carefully adjusted, and phase compensation using additional wave plates (not shown in Figure 2 in the main text or Supplementary Figures 12 and 13) between different spatial modes is applied. In our experiment, the fidelity for the investigation of the geometric phases is approximately $94.13 \pm 0.04\%$, and the fidelities for the investigation of flip-error immunity and phase-error immunity are approximately $97.91 \pm 0.03\%$ and $96.99 \pm 0.04\%$, respectively.

SUPPLEMENTARY NOTE 8. ERROR ESTIMATION

In our experiment, the statistics of each count is assumed to follow a Poisson distribution, which are determined by the subprogram of PoissonDistribution in Wolfram Mathematica 7.0. The values of each measurement quantity are then calculated from 50 randomly grouped counting sets, in which the error of the quantity is estimated by the square root of the corresponding variance. The counts for each measurement in our experiment is about 100K *counts/s*. As a result, the variances are small and the error bars are smaller than the size of the symbols in the figures. In our experiment, the phases are stable in the interferometers constructed by beam displacers. However, due to the fact that there would be maximal 12 beam displacers involved during the measurement, the misalignments of beam displacers would limit the fidelity to reconstruct the braiding matrixes and the final states.

SUPPLEMENTARY NOTE 9. MORE EXPERIMENTAL RESULTS

The six experimental initial states (after the first DE0) are shown in Supplementary Figure 9**a** and **d**. The corresponding final states (after the second DE0) in demonstrating the flip-error immunity and phase-error immunity are shown in Supplementary figure 9**b** and **e**, respectively. The black dots in the poles of the Bloch sphere represent the corresponding theoretical predictions with the states $|xxx\rangle$ (+**Z** direction), $\frac{1}{\sqrt{2}}(|xxx\rangle - i|\bar{x}\bar{x}\bar{x}\rangle)$ (-**Y** direction), $|\bar{x}\bar{x}\bar{x}\rangle$ (-**Z** direction), $\frac{1}{\sqrt{2}}(|xxx\rangle + |\bar{x}\bar{x}\bar{x}\rangle)$ (+**X** direction, $|0_{3s}\rangle$), $\frac{1}{\sqrt{2}}(|xxx\rangle + i|\bar{x}\bar{x}\bar{x}\rangle)$ (+**Y** direction), and $\frac{1}{\sqrt{2}}(|xxx\rangle - |\bar{x}\bar{x}\bar{x}\rangle)$ (-**X** direction, $|1_{3s}\rangle$), respectively. The final states after the flip-error operation and the phase-error operation are almost identical to the corresponding initial states, respectively. Supplementary Figure 9**c.** and **f.** show the comparison between the fidelities of the final states (dark yellow columns) and the theoretical prediction (identity, cyan columns) for the cases of flip-error protection and phase-error protection, respectively.

The output states of the first DE0 and DE1 in Figure 2 in the main text are shown in Supplementary Figure 10**a** and **c**, respectively. The fidelities, calculated as $(\mathrm{Tr}\sqrt{\sqrt{\rho_{\mathrm{e}}}\rho_{\mathrm{t}}\sqrt{\rho_{\mathrm{e}}}})^2$, of the corresponding output states are shown in Supplementary Figure 10**b** and **d**, where ρ_{e} (ρ_{t}) represents the experimentally reconstructed (theoretically predicted) density matrix. Supplementary Figure 10**e** shows the final states after the second DE0 and Supplementary Figure 10**f** shows the corresponding fidelities. The initial states in the meridian plane of

the Bloch sphere (Supplementary Figure 10**a**) is rotated counterclockwise to the equatorial plane (Supplementary Figure 10**e**), which clearly demonstrate the non-trivial statistics. The error bars for fidelities correspond to the counting statistics.

Supplementary Figure 11 shows experimental results for the four-mode measurement in investigating the geometric phases. Solid and open columns represent the theoretical and experimental results of the density matrices after DE2. The fidelities are shown in Supplementary Figure 11**j**, with the numbers from 1 to 9 representing the cases with the theoretical initial states of $|xxx\rangle$ (**a**), $\frac{3}{\sqrt{10}}|xxx\rangle + \frac{1}{\sqrt{10}}|\bar{x}\bar{x}\bar{x}\rangle$ (**b**), $\frac{2}{\sqrt{5}}|xxx\rangle + \frac{1}{\sqrt{5}}|\bar{x}\bar{x}\bar{x}\rangle$ (**c**), $\frac{1}{\sqrt{2}}(|xxx\rangle + |\bar{x}\bar{x}\bar{x}\rangle)$ (**d**), $\frac{1}{\sqrt{5}}|xxx\rangle + \frac{2}{\sqrt{5}}|\bar{x}\bar{x}\bar{x}\rangle$ (**e**), $\frac{1}{\sqrt{10}}|xxx\rangle + \frac{3}{\sqrt{10}}|\bar{x}\bar{x}\bar{x}\rangle$ (**f**), $|\bar{x}\bar{x}\bar{x}\rangle$ (**g**), $\frac{1}{\sqrt{2}}(|xxx\rangle + i|\bar{x}\bar{x}\bar{x}\rangle)$ (**h**), and $\frac{1}{\sqrt{2}}(|xxx\rangle - i|\bar{x}\bar{x}\bar{x}\rangle)$ (**i**), respectively.

SUPPLEMENTARY NOTE 10. EXPERIMENTAL SETUP FOR SIMULATING THE EXCHANGE OF MZMS

As shown in the Pre pane in Figure 2 in the main text, the polarization of a single photon is rotated using a half-wave plate (HWP), and the photon is then sent to a BD30 with a vertical beam displacement of 3.0 mm. Both output beams are further rotated using HWPs and sent to another BD30 with a horizontal beam displacement of 3.0 mm. There are now four output beams, which are further rotated using HWPs and pass through a BD60 with a horizontal beam displacement of 6.0 mm. We then obtain eight output beams, and the distances between neighboring beams are all equal to each other, which facilitates the measurement of interferences between different optical modes. The relative amplitudes of the eight beams are controlled using HWPs in the corresponding modes. The eight optical beams represent the basis of the eight-dimensional space of the systems, which are initially expressed in the basis of σ^x as $\{|xxx\rangle, |xx\bar{x}\rangle, |x\bar{x}x\rangle, |x\bar{x}\bar{x}\rangle, |\bar{x}xx\rangle, |\bar{x}x\bar{x}\rangle, |\bar{x}\bar{x}x\rangle, |\bar{x}\bar{x}\bar{x}\rangle\}$.

The precision of the dissipative evolution is theoretically dependent on the scale of the evolution time t. In our protocol using a polarization beam splitter (PBS), it is dependent on the ratio between the reflected and transmitted parts of the vertical polarization after the PBS, which can be higher than 500:1. As a result, the optical modes with vertical polarization are discarded directly after each dissipative evolution process (the reflection of photons with vertical polarizations is not shown in Figure 2 in the main text). For the first dissipative evoultion DE0 (the projection of $\exp(\sigma_2^x\sigma_3^x * t)\exp(\sigma_1^x\sigma_2^x * t)$), the normalized

output state would have the form of $|\phi_0\rangle = \alpha|xxx\rangle + \beta|\bar{x}\bar{x}\bar{x}\rangle$. To perform DE1, we must express the system located at site 1 in the basis of σ^z, which is implemented by BR1, which contains two HWPs and a BD60. Consider the dissipative evolution of H_2: two HWPs and a BD30 in BR2 are used to transform the state of the system located at site 1 back to σ^x. The following setup contains HWPs and a BD60 to transform the state of the system located at site 2 to σ^z. To transform the state of the system located at site 3 to σ^y, two BD30s and two HWPs with a quarter-wave plate (QWP) are used. An inverse base rotation is implemented in BR3 to transform the entire state back to σ^x to implement DE0 once again.

For the case of two-mode measurement (TM), the two spatial modes are denoted by H and V, respectively. The amplitudes of the two modes, which correspond to the amplitudes of the two polarizations, can be measured directly. For the measurement of R and D, the interference between these two modes is needed. By applying a HWP in the mode denoted by V, the spatial modes can be combined again using a BD30. The interference of the optical modes is transformed to the interference of polarizations, which can be measured using the standard polarization-analysis setup. For the four-mode measurement (FM) case, the four modes are denoted by HH, VH, HV and VV. Measurements of interference between the optical modes are carried out using a step-by-step process. For example, the measurement of RR is obtained by first combining HH and VH to obtain RH and then combining HV and VV to obtain RV. RH and RV are then further combined to obtain RR. As a result, the requirement of the 16 measurement configurations for the standard two-qubit-state tomography can be achieved.

To fully characterize the behavior of the entire setup, we experimentally performed full quantum process tomography [20, 21]. By expanding the output state $\mathcal{E}(\rho)$ with a complete set of basis $\hat{E}_m$ of the Pauli operators $\{I(\text{identity}), \sigma^x(X), \sigma^y(Y), \sigma^z(Z)\}$, the operation of the quantum process can be expressed as $\mathcal{E}(\rho) = \sum_{mn} \chi_{mn} \hat{E}_m \rho \hat{E}_n^\dagger$. The physical process $\mathcal{E}$ is completely and uniquely characterized by the 4-by-4 matrix χ [22]. For simulating the exchange of MZMs, the final state can be written as $\frac{1}{\sqrt{2}}(\alpha - i\beta)|xxx\rangle + \frac{1}{\sqrt{2}}(\beta - i\alpha)|\bar{x}\bar{x}\bar{x}\rangle$ when the initial state is $\alpha|xxx\rangle + \beta|\bar{x}\bar{x}\bar{x}\rangle$. The experimental χ-matrix for demonstrating non-trivial statistics is denoted by $\chi_{\rm n}^{\rm e}$, while $\chi_{\rm n}^{\rm t}$ represents the theoretical prediction. The fidelity of the experimental result $\chi_{\rm n}^{\rm e}$ is approximately $94.13 \pm 0.04\%$, which can be calculated from $(\mathrm{Tr}\sqrt{\sqrt{\chi_{\rm n}^{\rm e}}\chi_{\rm n}^{\rm t}\sqrt{\chi_{\rm n}^{\rm e}}})^2$ [22]. The experimental χ-matrix for demonstrating the immunity against flip errors is denoted by $\chi_{\rm f}^{\rm e}$. $\chi_{\rm f}^{\rm t}$ represents the theoretical prediction, which corresponds to

identity. The experimental fidelity of $\chi_{\rm f}^{\rm e}$, which can be calculated from $(\mathrm{Tr}\sqrt{\sqrt{\chi_{\rm f}^{\rm e}}\chi_{\rm f}^{\rm t}\sqrt{\chi_{\rm f}^{\rm e}}})^2$, is approximately $97.91 \pm 0.03\%$. Similarly, the experimental χ-matrix for demonstrating the immunity against phase errors is denoted by $\chi_{\rm p}^{\rm e}$. $\chi_{\rm p}^{\rm t}$ represents the theoretical prediction, which corresponds to identity. The experimental fidelity of $\chi_{\rm p}^{\rm e}$, which can be calculated from $(\mathrm{Tr}\sqrt{\sqrt{\chi_{\rm p}^{\rm e}}\chi_{\rm p}^{\rm t}\sqrt{\chi_{\rm p}^{\rm e}}})^2$, is approximately $96.99 \pm 0.04\%$. Optical systems have been used to simulate a three-spin Ising ring [23] and the XY spin model Hamiltonian [24]. Our experiment simulates a spin chain that is equal to the Kitaev chain and investigates the properties of MZMs in this model. This is completely different from the work simulating the dynamics of a charged Majorana particle by employing a tailored waveguide chip [25].

SUPPLEMENTARY NOTE 11. EXPERIMENTAL SETUP FOR DEMONSTRATING LOCAL NOISES IMMUNITY

The experimental setup for the investigation of the flip-error immunity is shown in Supplementary Figure 12. Similar to the case for probing the geometric phases, the input state is prepared by the setup denoted by Pre. The output state after the dissipative evolution DE0 ($\alpha|xxx\rangle + \beta|\bar{x}\bar{x}\bar{x}\rangle$) is then treated as the initial state. The flip-error operation is realized by six HWPs, a BD30 and a BD60, in which the initial two optical spatial modes are transformed into eight spatial modes with the state representation of $|xxx\rangle$ and $|\bar{x}\bar{x}\bar{x}\rangle$ (with the operation of $\sigma_1^x\sigma_2^x$), $|x\bar{x}x\rangle$ and $|\bar{x}x\bar{x}\rangle$ (with the operation of $-i\sigma_1^x\sigma_2^y$), $|\bar{x}xx\rangle$ and $|x\bar{x}\bar{x}\rangle$ (with the operation of $i\sigma_1^y\sigma_2^x$), $|\bar{x}\bar{x}x\rangle$ and $|xx\bar{x}\rangle$ (with the operation of $\sigma_1^y\sigma_2^y$). Two of the eight output modes ($|xxx\rangle$ and $|\bar{x}\bar{x}\bar{x}\rangle$) are preserved after the second DE0. The two-mode (TM) measurement setup is used to reconstruct the initial and final states, in which the states of spatial information are translated into polarization information. A quarter-wave plate (QWP), a half-wave plate (HWP) and a polarization beam splitter (PBS) are used for polarization analysis. Photons are finally detected by single-photon detectors (SPDs).

Supplementary Figure 13 shows the experimental setup for the investigation of the phase-error immunity, which is similar to Supplementary Figure 8. For the perturbation operator $\frac{1}{2}(\sigma_1^z + 1)$, the initial two-mode states transform into four-mode states, two of which remain unchanged, while two are rotated by σ_1^z. HWPs and a BD30 are used to implement the perturbation, using a setup similar to that shown in BR1. The basis of the four-mode states in BR1 (to express the first qubit in σ^z) are represented by $\{|zxx\rangle, |\bar{z}xx\rangle, |z\bar{x}\bar{x}\rangle, |\bar{z}\bar{x}\bar{x}\rangle\}$,

while the basis of the four-mode states in the case of perturbation are represented by $\{|xxx\rangle, |\bar{x}\bar{x}\bar{x}\rangle, |\bar{x}xx\rangle, |x\bar{x}\bar{x}\rangle\}$. After the second DE0, only the terms associated to $|xxx\rangle$ and $|\bar{x}\bar{x}\bar{x}\rangle$ are preserved.

SUPPLEMENTARY REFERENCES

[1] Kitaev, A. Y. Unpaired Majorana fermions in quantum wires. *Phys.-Usp.* **44**, 131-136 (2001).

[2] Alicea, J., Oreg, Y., Refael, G., Oppen, F. & Fisher, M. P. A. Non-Abelian statistics and topological quantum information processing in 1D wire networks. *Nature Phys.* **7**, 412-417 (2011).

[3] Mezzacapo, A., Casanova, J., Lamata, L. & Solano, E. Topological qubits with Majorana fermions. *New J. Phys.* **15**, 033005 (2013).

[4] Mi, S., Pikulin, D. I., Wimmer, M. & Beenakker, C. W. J. Proposal for the detection and braiding of Majorana fermions in a quantum spin Hall insulator. *Phys. Rev. B* **87**, 241405(R) (2013).

[5] Poddubny, A., Miroshnichenko, A., Slobozhanyuk, A. & Kivshar, Y. Topological Majorana states in zigzag chains of plasmonic nanoparticles. *ACS Photon.* **1**, 101-105 (2014).

[6] Lahtinen V., & Pachos J. K., Non-Abelian statistics as a Berry phase in exactly solvable models, *New J. Phys.* **11** 093027 (2009).

[7] Kapit E.,Ginsparg P., & Mueller E., Non-Abelian Braiding of Lattice Bosons. *Phys. Rev. Lett.* **108**, 066802 (2012).

[8] Xu, J.-S., *et al.*, Demon-like algorithmic quantum cooling and its realization with quantum optics *Nature Photon.* **8**, 113-118 (2014).

[9] Fendally, P. Parafermionic edge zero modes in Zn-invariant spin chains. *J. Stat. Mech.* **11**, P11020 (2012).

[10] Kitaex, A. & Laumann, C. 'Topological phases and quantum computation' in Les Houches Summer School 'Exact methods in low-dimensional physics and quantum computing'. (2008).

[11] Suzuki M. Generalized Trotter's formula and systematic approximants of exponential operators and inner derivations with applications to many-body problems. *Commun. Math. Phys.* **51**, 183-190 (1976).

[12] Bargmann, V. Note on Wigner's theorem on symmetry operations. *J. Math. Phys.* **5**, 862-868 (1964).

[13] Simon, R. & Mukunda, N. Bargmann invariant and the geometric of the Güoy effect. *Phys. Rev. Lett.* **70**, 880-883 (1993).

[14] Rabei, E. M., Arvind, Mukunda N. & Simon, R. Bargmann invariants and geometric phase: A generalized connection. *Phys. Rev. A* **60**, 3397-3409 (1993)

[15] Aharonov, Y. & Anandan, J. Phase change during a cyclic quantum evolution. *Phys. Rev. Lett.* **58**, 1593-1596 (1987).

[16] Pancharatnam, S., Generalized theory of interference, and its applications. *Proc.Ind. Acad. Sci. A* **44**, 247-262 (1956).

[17] Xue Z.-Y., Detecting non-Abelian statistics of Majorana fermions in quantum nanowire networks. *JETP Lett.* **94**, 213-216 (2011).

[18] Vidal, G., Classical simulation of infinite-size quantum lattice systems in one spatial dimension. *Phys. Rev. Lett.* **98**, 070201 (2007).

[19] Kitagawa, T. *et al.*, Observation of topologically protected bound states in photonic quantum walks. *Nat. Commun.* **3** , 882 (2012).

[20] Chuang, I. L. & Nielsen, M. A. Prescrition for experimental determination of the dynamics of a quantum black box. J. Mod. Opt. **44**, 2455-2467 (1997).

[21] Poyatos, J. F., Cirac, J. I. & Zoller, P. Complete characterization of a quantum process: the two-bit quantum gate. *Phys. Rev. Lett.* **78**, 390-393 (1997).

[22] O'Brien, J. L. *et al.*, Quantum process tomography of a Controlled-NOT gate. *Phys. Rev. Lett.* **93**, 080502 (2004).

[23] Orieux, A., Boutari, J., Barbieri, M., Paternostro, M. & Mataloni, P., Experimental linear-optics simulation of multipartite non-locality in the ground state of a quantum Ising ring. *Sci. Rep.* **4**, 7184 (2014).

[24] Barz, S. *et al.*, Linear-Optical Generation of Eigenstates of the Two-Site XY Model. *Phys. Rev. X* **5**, 021010 (2015).

[25] Keil, R. *et al.*, Optical simulation of charge conservation violation and Majorana dynamics. *Optica* **2**, 454-459 (2015).
